# m^6^A regulator-mediated methylation modification patterns and tumor microenvironment infiltration characterization in gastric cancer

**DOI:** 10.1186/s12943-020-01170-0

**Published:** 2020-03-12

**Authors:** Bo Zhang, Qiong Wu, Ben Li, Defeng Wang, Lei Wang, You Lang Zhou

**Affiliations:** 1 Research Center of Clinical Medicine, Affiliated Hospital of Nantong University, Nantong, 226001 Jiangsu Province China; 2 Medical School of Nantong University, Nantong, 226001 Jiangsu Province China; 3 Department of Cardiothoracic Surgery, Affiliated Hospital of Nantong University, Nantong, 226001 Jiangsu Province China; 4 State Key Laboratory of Microbial Metabolism, School of Life Sciences and Biotechnology, Shanghai Jiao Tong University, Shanghai, 200240 China

**Keywords:** m^6^A, Tumor microenvironment, Stroma, Immunotherapy, Mutation burden

## Abstract

**Background:**

The epigenetic regulation of immune response has been demonstrated in recent studies. Nonetheless, potential roles of RNA N6-methyladenosine (m^6^A) modification in tumor microenvironment (TME) cell infiltration remain unknown.

**Methods:**

We comprehensively evaluated the m^6^A modification patterns of 1938 gastric cancer samples based on 21 m^6^A regulators, and systematically correlated these modification patterns with TME cell-infiltrating characteristics. The m6Ascore was constructed to quantify m^6^A modification patterns of individual tumors using principal component analysis algorithms.

**Results:**

Three distinct m^6^A modification patterns were determined. The TME cell-infiltrating characteristics under these three patterns were highly consistent with the three immune phenotypes of tumors including immune-excluded, immune-inflamed and immune-desert phenotypes. We demonstrated the evaluation of m^6^A modification patterns within individual tumors could predict stages of tumor inflammation, subtypes, TME stromal activity, genetic variation, and patient prognosis. Low m6Ascore, characterized by increased mutation burden and activation of immunity, indicated an inflamed TME phenotype, with 69.4% 5-year survival. Activation of stroma and lack of effective immune infiltration were observed in the high m6Ascore subtype, indicating a non-inflamed and immune-exclusion TME phenotype, with poorer survival. Low m6Ascore was also linked to increased neoantigen load and enhanced response to anti-PD-1/L1 immunotherapy. Two immunotherapy cohorts confirmed patients with lower m6Ascore demonstrated significant therapeutic advantages and clinical benefits.

**Conclusions:**

This work revealed the m^6^A modification played a nonnegligible role in formation of TME diversity and complexity. Evaluating the m^6^A modification pattern of individual tumor will contribute to enhancing our cognition of TME infiltration characterization and guiding more effective immunotherapy strategies.

**Graphical abstract:**

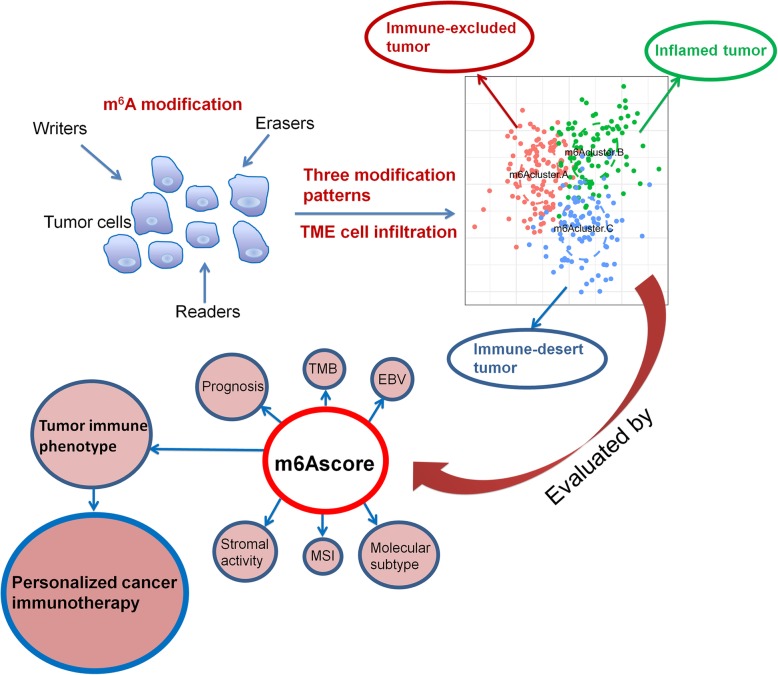

## Introduction

In all living organisms, as the third layer of epigenetics, more than 150 RNA modifications including 5-methylcytosine (m^5^C), N6-methyladenosine (m^6^A) and N1-methyladenosine (m^1^A) have been identified [1, 2]. Among these modifications, m^6^A RNA methylation, which are widely found in the mRNA, lncRNA as well as miRNA, is recognized as the most prominent and abundant form of internal modifications in eukaryotic cells, of whose abundance account for 0.1–0.4% total adenosine residues [3–5]. Similar to the modification of DNA and protein, m^6^A modification is a kind of dynamic reversible process in mammalian cells, which is regulated by methyltransferases, demethylases and binding proteins, also known as “writers”, “erasers” and “readers” [6]. The formation process of m^6^A methylation is catalyzed by methyltransferases consisting of RBM15, ZC3H13, METTL3, METTL14, WTAP and KIAA1429, while the removal process is mediated by demethylases including FTO and ALKBH5. In addition, a group of specific RNA-binding proteins composed of YTHDF1/2/3, YTHDC1/2, HNRNPA2B1, LRPPRC, FMR1 and so on can recognize m^6^A motif, thus affecting m^6^A functions [7, 8]. The in-depth understanding of these regulators would help reveal the role and mechanism of m^6^A modification in post-transcriptional regulation. It has been reported that the m^6^A regulators play a crucial role in a variety of biological functions in vivo [9–11]. Increasing evidence demonstrated that dysregulated expression and genetic changes of m^6^A regulators were correlated with the disorders of multiple biological process including dysregulate cell death and proliferation, developmental defects, tumor malignant progression, impaired self-renewal capacity, and immunomodulatory abnormality [12–14].

Immunotherapy represented by immunological checkpoint blockade (ICB, PD-1/L1 and CTLA-4) has demonstrated astounding clinical efficacy in a small percentage of patients with durable responses. Unfortunately, the majority of patients experienced minimal or no clinical benefit, far from a met clinical need [15]. Traditionally, the tumor progression has been considered as a multi-step process that only involves the genetic and epigenetic variation in tumor cells. However, numerous studies have shown that the microenvironment in which tumor cells depend for growth and survival also play a crucial role in the tumor progression. The tumor part was composed of a complex tumor microenvironment (TME) that not only contained cancer cells but also stromal cells such as resident fibroblasts (cancer associated fibroblast; CAF) and macrophages, and distant recruited cells such as infiltrating immune cells (myeloid cells and lymphocytes), bone marrow-derived cells (BMDCs) such as endothelial progenitor and hematopoietic progenitor cells, secreted factors such as cytokines, chemokines, growth factors, and new blood vessels. Of these, five distinct myeloid populations including tumor-associated macrophages (TAM), tumor-associated neutrophils (TANs), dendritic cells, myeloid-derived suppressor cells (MDSCs) and Tie2-expressing monocytes comprised the tumor-associated myeloid cells (TAMCs) [16]. Cancers cells elicited multiple biological behavior changes through direct and indirect interactions with other TME components such as inducing proliferation and angiogenesis, inhibiting apoptosis, avoiding hypoxia as well as inducing immune tolerance. As the understanding of the diversity and complexity of tumor microenvironment has deepened, emerging evidence reveals its critical role in the tumor progression, immune escape, and its effect on response to immunotherapy. Predicting the response to ICB based on the characterization of TME cell infiltration is a key procedure on increasing the success of existing ICBs and exploiting novel immunotherapeutic strategies [17, 18]. Therefore, by comprehensively parsing the TME landscape heterogeneity and complexity, different tumor immune phenotypes are likely to be identified, and the abilities of guiding and predicting immunotherapeutic responsiveness would also improve. Additionally, the promising biomarkers could be revealed, which will prove highly effective in recognizing patients’ response to immunotherapy and will benefit the search for new therapeutic targets [19, 20].

Recently, several studies have revealed the special correlation between TME infiltrating immune cells and m^6^A modification, which can’t be explained via RNA degradation mechanism. Dali et al. reported that binding of YTHDF1 to the transcripts encoding lysosomal proteases modified by m^6^A methylation improved the translational efficiency of lysosomal cathepsins in dendritic cells (DCs), while suppression of cathepsins in DC significantly strengthened its ability to cross-present tumor antigens, which in turn enhanced tumor infiltrating CD8+ T cell antitumor response. And YTHDF1 inhibition also improved the therapeutic efficacy of anti-PD-L1 blockade [21]. The study of Huamin et al. revealed that METTL3-mediated m^6^A modification promoted the activation and maturation of DCs. Declining expression of co-stimulatory molecules CD80 and CD40 resulted by METTL3 specific depletion reduced the ability of stimulating T cell activation. And down-regulation of Tirap inhibited the transmission of the TLR4/NF-κB signaling pathway and decreased the secretion of pro-inflammatory cytokines [22]. In addition, some studies have focused on the intrinsic oncogenic pathways induced by dysregulated expression and genomic variation of m^6^A regulators. For example, Qiang et al. found that METTL3 overexpression promote gastric cancer (GC) malignant progression and liver metastasis through angiogenesis and glycolysis pathway [23].

However, the above studies have necessarily been confined to only one or two m^6^A regulators and cell types owing to technical limitations, while the antitumor effect is characterized by numerous tumor suppressor factors that interact in a highly coordinated manner. Therefore, comprehensive recognizing the TME cell infiltration characterizations mediated by multiple m^6^A regulators will contribute to enhancing our understanding of TME immune regulation. In this study, we integrated the genomic information of 1938 gastric cancer samples to comprehensively evaluate the m^6^A modification patterns, and correlated the m^6^A modification pattern with the TME cell-infiltrating characteristics. We revealed three distinct m^6^A modification patterns, and surprisingly found that the TME characteristics under these three patterns were highly consistent with the immune-excluded phenotype, immune-inflamed phenotype and immune-desert phenotype, respectively, suggesting the m^6^A modification played a nonnegligible role in shaping individual tumor microenvironment characterizations. For that, we established a set of scoring system to quantify the m^6^A modification pattern in individual patients.

## Methods

### Gastric cancer dataset source and preprocessing

The workflow of our study was shown in Figure S1A. Public gene-expression data and full clinical annotation were searched in Gene-Expression Omnibus (GEO) and the Cancer Genome Atlas (TCGA) database. Patients without survival information were removed from further evaluation. In total, 7 eligible GC cohorts (GSE15459, GSE34942, GSE57303, GSE62254/ACRG, GSE84437, GSE26253 and TCGA-STAD (The Cancer Genome Atlas-Stomach Adenocarcinoma)) were gathered in this study for further analysis. For microarray data from Affymetrix®, we downloaded the raw “CEL” files and adopted a robust multiarray averaging method with the affy and simpleaffy packages to perform background adjustment and quantile normalization. For microarray data from other platforms, the normalized matrix files were directly downloaded. As to datasets in TCGA, RNA sequencing data (FPKM value) of gene expression were downloaded from the Genomic Data Commons (GDC, https://portal.gdc.cancer.gov/) using the R package TCGAbiolinks [24], which was specifically developed for integrative analysis with GDC data [24]. Then FPKM values were transformed into transcripts per kilobase million (TPM) values. Batch effects from non-biological technical biases were corrected using the “ComBat” algorithm of sva package. The baseline information of all eligible GC datasets was summarized in Table S1. The somatic mutation data was acquired from TCGA database. The GSE62717 dataset from ACRG cohort was downloaded for Copy Number Variation (CNV) analysis. Data were analyzed with the R (version 3.6.1) and R Bioconductor packages.

### Unsupervised clustering for 21 m^6^A regulators

Owing to the few m^6^A regulators detected by Illumina HumanRef-8 WG-DASL v3.0 platform, we did not include GSE26253 cohort for clustering analysis. A total of 21 regulators were extracted from five integrated GEO datasets for identifying different m^6^A modification patterns mediated by m^6^A regulators. These 21 m^6^A regulators included 8 writers (METTL3, METTL14, RBM15, RBM15B, WTAP, KIAA1429, CBLL1, ZC3H13), 2 erasers (ALKBH5, FTO) and 11 readers (YTHDC1, YTHDC2, YTHDF1, YTHDF2, YTHDF3, IGF2BP1, HNRNPA2B1, HNRNPC, FMR1, LRPPRC, ELAVL1). Unsupervised clustering analysis was applied to identify distinct m^6^A modification patterns based on the expression of 21 m^6^A regulators and classify patients for further analysis. The number of clusters and their stability were determined by the consensus clustering algorithm [25]. We used the ConsensuClusterPlus package to perform the above steps and 1000 times repetitions were conducted for guaranteeing the stability of classification [26].

### Gene set variation analysis (GSVA) and functional annotation

To investigate the difference on biological process between m^6^A modification patterns, we performed GSVA enrichment analysis using “GSVA” R packages. GSVA, in a non-parametric and unsupervised method, is commonly employed for estimating the variation in pathway and biological process activity in the samples of an expression dataset [27]. The gene sets of “c2.cp.kegg.v6.2.symbols” were downloaded from MSigDB database for running GSVA analysis. Adjusted P with value less than 0.05 was considered as statistically significance. The clusterProfiler R package was used to perform functional annotation for m^6^A-related genes, with the cutoff value of FDR < 0.05.

### Estimation of TME cell infiltration

We used the ssGSEA (single-sample gene-set enrichment analysis) algorithm to quantify the relative abundance of each cell infiltration in the GC TME. The gene set for marking each TME infiltration immune cell type was obtained from the study of Charoentong, which stored various human immune cell subtypes including activated CD8 T cell, activated dendritic cell, macrophage, natural killer T cell, regulatory T cell and so on (Table S2) [28, 29]. The enrichment scores calculated by ssGSEA analysis were utilized to represent the relative abundance of each TME infiltrating cell in each sample.

### Identification of differentially expressed genes (DEGs) between m^6^A distinct phenotypes

To identify m^6^A-related genes, we classified patients into three distinct m^6^A modification patterns based on the expression of 21 m^6^A regulators. The empirical Bayesian approach of limma R package was applied to determine DEGs between different modification patterns [30]. The significance criteria for determining DEGs was set as adjusted *P* value < 0.001.

### Generation of m^6^A gene signature

To quantify the m^6^A modification patterns of individual tumor, we constructed a set of scoring system to evaluate the m^6^A modification pattern of individual patients with gastric cancer—the m^6^A gene signature, and we termed as m6Ascore. The procedures for establishment of m^6^A gene signature were as follows:

The DEGs identified from different m6Aclusters were firstly normalized among all ACRG samples and the overlap genes were extracted. The patients were classified into several groups for deeper analysis by adopting unsupervised clustering method for analyzing overlap DEGs. The consensus clustering algorithm was utilized for defining the number of gene clusters as well as their stability. Then, we performed the prognostic analysis for each gene in the signature using univariate Cox regression model. The genes with the significant prognosis were extracted for further analysis. We then conducted principal component analysis (PCA) to construct m^6^A relevant gene signature. Both principal component 1 and 2 were selected to act as signature scores. This method had advantage of focusing the score on the set with the largest block of well correlated (or anticorrelated) genes in the set, while down-weighting contributions from genes that do not track with other set members. We then define the m6Ascore using a method similar to GGI [31, 32]:

$$ m6 Ascore=\sum \left( PC{1}_i+ PC{2}_i\right) $$

where i is the expression of m^6^A phenotype-related genes.

### Correlation between m^6^A gene signature and other related biological processes

Mariathasan et al. constructed a set of gene sets that stored genes associated with some biological processes, including (1) immune-checkpoint; (2) antigen processing machinery; (3) CD8 T-effector signature; (4) epithelial-mesenchymal transition (EMT) markers including EMT1, EMT2 and EMT3; (5) Angiogenesis signature; (7) pan-fibroblast TGFb response signature (Pan-F-TBRS); (8) WNT targets; (9) DNA damage repair; (10) mismatch repair; (11) Nucleotide excision repair; (12) DNA replication; (13) Antigen processing and presentation [33–35]. We them performed a correlation analysis to further reveal the association between m^6^A gene signature and some related biological pathways.

### Collection of immune-checkpoint blockade genomic and clinical information

We performed a systematical search for the immune checkpoint blockade gene expression profiles, which could be publicly obtained and reported with complete clinical information. Two immunotherapeutic cohorts were finally included in our study: advanced urothelial cancer with intervention of atezolizumab, an anti-PD-L1 antibody (IMvigor210 cohort) [33], and metastatic melanoma treated with pembrolizumab, an anti-PD-1 antibody (GSE78220 cohort downloaded from GEO) [36]. For IMvigor210 cohort, based on the Creative Commons 3.0 License, the complete expression data and detailed clinical annotations could be obtained from http://research-pub.Gene.com/imvigor210corebiologies. The raw count data were normalized by the DEseq2 R package and then the count value was transformed into the TPM value. For GSE78220 cohort, after standardization using limma package, the FPKM data of gene expression profiles was also converted to the more comparable TPM value among samples.

### Statistical analysis

Correlations coefficients between the TME infiltrating immune cells and expression of m^6^A regulators were computed by Spearman and distance correlation analyses. One-way ANOVA and Kruskal-Wallis tests were used to conduct difference comparisons of three or more groups [37]. On the basis of the correlation between m6Ascore and patients’ survival, the cut-off point of each dataset subgroup was determined using the survminer R package. The “surv-cutpoint” function, which repeatedly tested all potential cut points in order for finding the maximum rank statistic, was applied to dichotomize m6Ascore, and then patients were divided into high and low m6Ascore groups based on the maximally selected log-rank statistics to decrease the batch effect of calculation. The survival curves for the prognostic analysis were generated via the Kaplan-Meier method and log-rank tests were utilized to identify significance of differences. We adopted a univariate Cox regression model to calculate the hazard ratios (HR) for m^6^A regulators and m^6^A phenotype-related genes. The independent prognostic factors were ascertained through a multivariable Cox regression model. Patients with detailed clinical data were eligible for final multivariate prognostic analysis. The forestplot R package was employed to visualize the results of multivariate prognostic analysis for m6Ascore in ACRG cohort and TCGA-STAD cohort. The specificity and sensitivity of m6Ascore were assessed through receiver operating characteristic (ROC) curve, and the area under the curve (AUC) were quantified using pROC R package. The waterfall function of maftools package was used to present the mutation landscape in patients with high and low m6Ascore subtype in TCGA-STAD cohort. The R package of RCircos was adopted to plot the copy number variation landscape of 21 m^6^A regulators in 23 pairs of chromosomes [38]. All statistical *P* value were two-side, with *p* < 0.05 as statistically significance. All data processing was done in R 3.6.1 software.

## Results

### Landscape of genetic variation of m^6^A regulators in gastric cancer

**Fig. 1:**
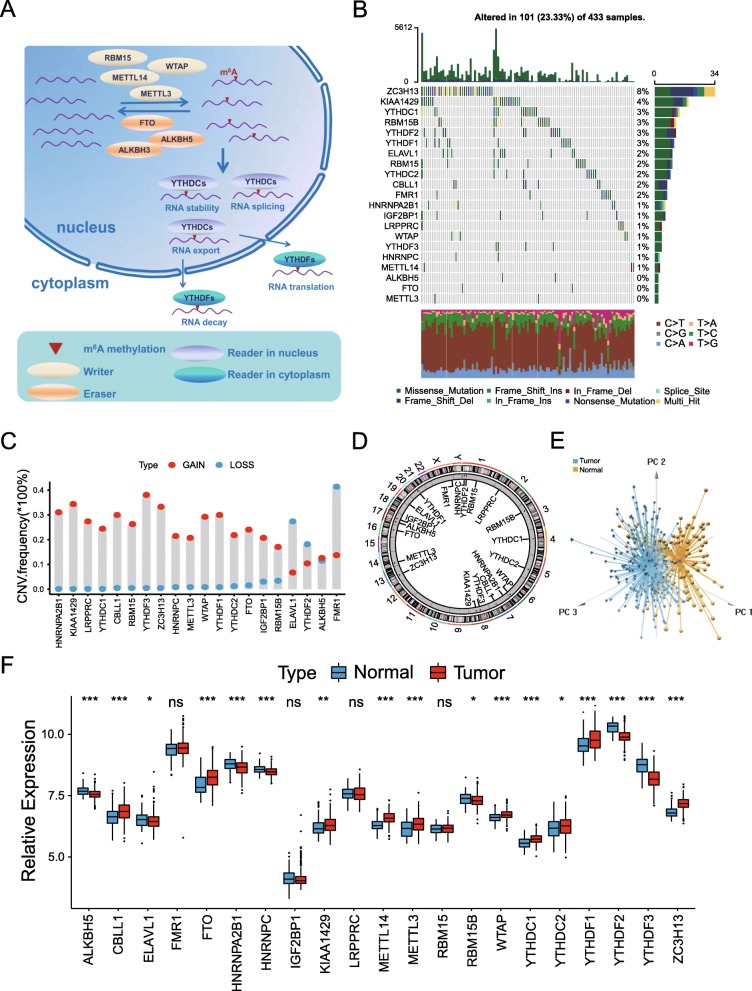
Landscape of genetic and expression variation of m^6^A regulators in gastric cancer. **a** Summary of the dynamic reversible process of m^6^A RNA methylation mediated by regulators (“writers”, “erasers” and “readers”) and their potential biological functions for RNA. **b** The mutation frequency of 21 m^6^A regulators in 433 patients with gastric cancer from TCGA-STAD cohort. Each column represented individual patients. The upper barplot showed TMB, The number on the right indicated the mutation frequency in each regulator. The right barplot showed the proportion of each variant type. The stacked barplot below showed fraction of conversions in each sample. **c** The CNV variation frequency of m^6^A regulators in GSE62717 cohort. The height of the column represented the alteration frequency. The deletion frequency, blue dot; The amplification frequency, red dot. **d** The location of CNV alteration of m^6^A regulators on 23 chromosomes using GSE62717 cohort. **e** Principal component analysis for the expression profiles of 21 m^6^A regulators to distinguish tumors from normal samples in GSE2269 cohort. Two subgroups without intersection were identified, indicating the tumors and normal samples were well distinguished based on the expression profiles of m^6^A regulators. Tumors were marked with blue and normal samples marked with yellow. **f** The expression of 21 m^6^A regulators between normal tissues and gastric tissues. Tumor, red; Normal, blue. The upper and lower ends of the boxes represented interquartile range of values. The lines in the boxes represented median value, and black dots showed outliers. The asterisks represented the statistical *p* value (*P < 0.05; **P < 0.01; ***P < 0.001)

A total of 21 m^6^A regulators including 8 writers, 2 erasers and 11 readers were finally identified in this study. Figure 1a summarized the dynamic reversible process of m6A RNA methylation mediated by regulators as well as their potential biological functions for RNA. We first summarized the incidence of copy number variations and somatic mutations of 21 m^6^A regulators in GC. Among 433 samples, 101 experienced mutations of m^6^A regulators, with frequency 23.33%. It was found that the ZC3H13 exhibited the highest mutation frequency followed by KIAA1429, while both demethylases (FTO and ALKBH5) as well as METTL3 did not show any mutations in GC samples (Fig. 1b). Further analyses revealed a significant mutation co-occurrence relationship between ELAVL1 and KIAA1429, YTHDF1 and ZC3H13, along with RBM15 and YTHDC1 (Figure S1B). The investigation of CNV alteration frequency showed a prevalent CNV alteration in 21 regulators and most were focused on the amplification in copy number, while ELAVL1, YTHDF2 and FMR1 had a widespread frequency of CNV deletion (Fig. 1c). The location of CNV alteration of m^6^A regulators on chromosomes was shown in Fig. 1d. Based on the expression of these 21 m^6^A regulators, we could completely distinguished GC samples from normal samples (Fig. 1e). To ascertain whether the above genetic variations influenced the expression of m^6^A regulators in GC patients, we investigated the mRNA expression levels of regulators between normal and GC samples, and found that the alterations of CNV could be the prominent factors resulting in perturbations on the m^6^A regulators expression. Compared to normal gastric tissues, m^6^A regulators with amplificated CNV demonstrated markedly higher expression in GC tissues (e.g. CBLL1 and FTO), and vice versa (e.g. ELAVL1 and YTHDF2) (Fig. 1c and f). The above analyses presented the highly heterogeneity of genetic and expressional alteration landscape in m^6^A regulators between normal and GC samples, indicating that the expression imbalance of m^6^A regulators played a crucial role in the GC occurrence and progression.

### m^6^A methylation modification patterns mediated by 21 regulators

**Fig. 2:**
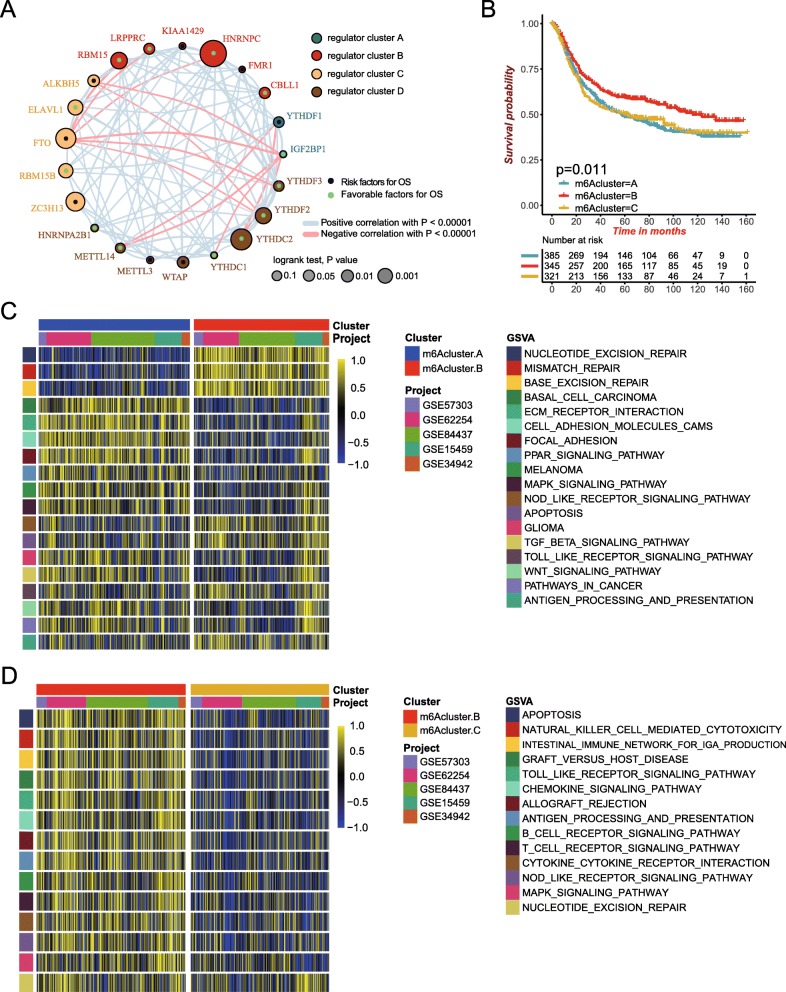
Patterns of m^6^A methylation modification and biological characteristics of each pattern. **a** The interaction between m^6^A regulators in gastric cancer. The circle size represented the effect of each regulator on the prognosis, and the range of values calculated by Log-rank test was *p* < 0.001, *p* < 0.01, *p* < 0.05 and *P* < 0.1, respectively. Green dots in the circle, risk factors of prognosis; Black dots in the circle, protective factors of prognosis. The lines linking regulators showed their interactions, and thickness showed the correlation strength between regulators. Negative correlation was marked with blue and positive correlation with red. The regulator cluster A-D was marked with blue, red, yellow and brown, respectively. **b** Survival analyses for the three m^6^A modification patterns based on 1051 patients with gastric cancer from five GEO cohorts (GSE15459, GSE34942, GSE57303, GSE62254/ACRG and GSE84437) including 389 cases in m6Acluster-A, 348 cases in m6Acluster-B, and 322 cases in m6Acluster-C. Kaplan-Meier curves with Log-rank *p* value 0.011 showed a significant survival difference among three m6A modification patterns. The m6Acluster B showed significantly better overall survival than the other two m6Acluster. **c-d** GSVA enrichment analysis showing the activation states of biological pathways in distinct m^6^A modification patterns. The heatmap was used to visualize these biological processes, and yellow represented activated pathways and blue represented inhibited pathways. The gastric cancer cohorts were used as sample annotations. **c** m6Acluster A vs m6Acluster B; **d** m6Acluster B vs m6Acluster C

Five GEO datasets with available OS data and clinical information (GSE15459, GSE34942, GSE57303, GSE62254/ACRG and GSE84437, Table S1) were enrolled into one meta-cohort. A univariate Cox regression model revealed the prognostic values of 21 m^6^A regulators in patients with gastric cancer (Figure S1C). The comprehensive landscape of m^6^A regulator interactions, regulator connection and their prognostic significance for GC patients was depicted with the m^6^A regulator network (Fig. 2a and Table S3). We found that not only the m^6^A regulators in the same functional category presented a remarkably correlation in expression, but also a significant correlation was shown among writers, erasers, and readers. We also demonstrated that whether tumors with a high writer gene expression exhibits a low eraser gene expression actually depended on the different writer and eraser genes (Figure S2A-S2H). It was found that tumors with a high expression of writer genes (WATP and RBM15) showed a low expression of eraser gene FTO, while the high expression of WATP and RBM15 did not affect the expression of another eraser gene ALKBH5 (Figure S2A-S2B). Tumors with a high expression of writer gene METTL14, METTL3, KIAA1429 and ZC3H13 showed a high expression of eraser gene FTO, and METTL14, METTL3 and ZC3H13 also did not interfere with ALKBH5 expression, while KIAA1429 shared a common trend in gene expression with ALKBH5. In addition, the change of RBM15B expression did not affect the expression of these two eraser genes (Figure S2C-S2H). Considering the relatively higher mutation frequency of writer gene ZC3H13, we analyzed the difference in expression of eraser genes between ZC3H13-mutant and wild types. Of these, ALKBH5 was significantly up-regulated in ZC3H13-mutant tumors compared to wild-type tumors, while FTO was significantly down-regulated (Figure S2I**).**

The above results indicated that cross-talk among the regulators of writers, readers, and erasers may play critical roles in the formation of different m^6^A modification patterns and TME cell-infiltrating characterization between individual tumors.

The R package of ConsensusClusterPlus was used to classify patients with qualitatively different m^6^A modification patterns based on the expression of 21 m^6^A regulators, and three distinct modification patterns were eventually identified using unsupervised clustering, including 389 cases in pattern A, 348 cases in pattern B and 322 cases in pattern C. We termed these patterns as m6Acluster A-C, respectively (Figure S2J and Table S4). Prognostic analysis for the three main m^6^A modification subtypes revealed the particularly prominent survival advantage in m6Acluster-B modification pattern (Fig. 2b).

### TME cell infiltration characteristics in distinct m^6^A modification patterns

**Fig. 3:**
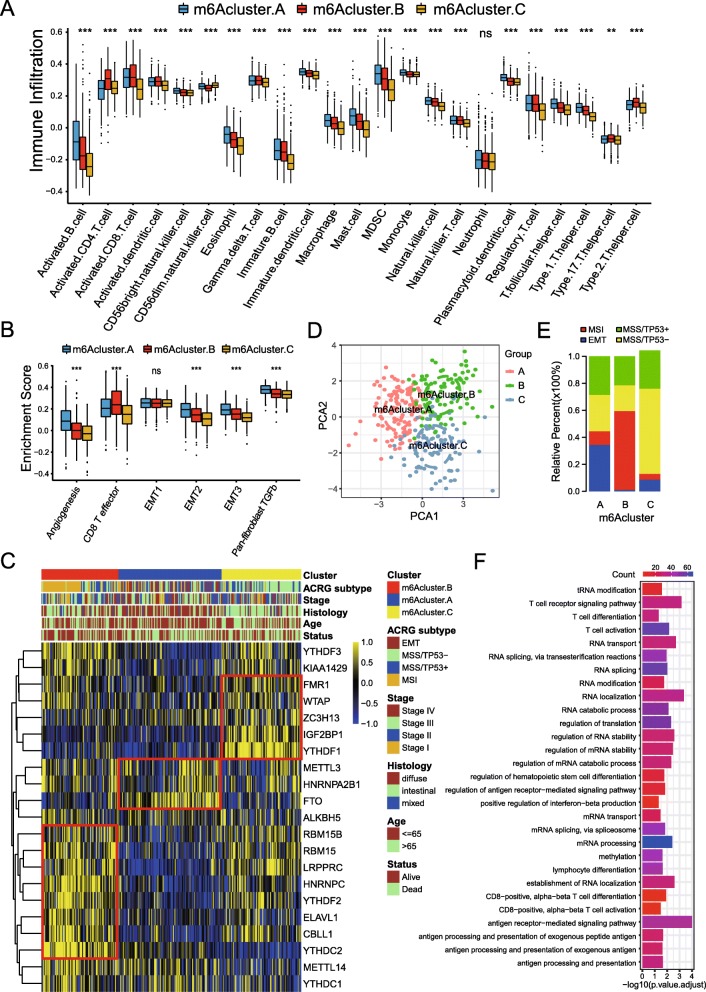
TME cell infiltration characteristics and transcriptome traits in distinct m^6^A modification patterns. **a** The abundance of each TME infiltrating cell in three m^6^A modification patterns. The upper and lower ends of the boxes represented interquartile range of values. The lines in the boxes represented median value, and black dots showed outliers. The asterisks represented the statistical *p* value (*P < 0.05; **P < 0.01; ***P < 0.001). **b** Differences in stroma-activated pathways including EMT, TGF beta and angiogenesis pathways among three distinct m^6^A modification patterns. The statistical differences among three modification patterns was tested by the one-way ANOVA test. The asterisks represented the statistical *p* value (*P < 0.05; **P < 0.01; ***P < 0.001). **c** Unsupervised clustering of 21 m^6^A regulators in the ACRG gastric cancer cohort. The m6Acluster, ACRG molecular subtypes, tumor stage, survival status and age were used as patient annotations. Yellow represented high expression of regulators and blue represented low expression. **d** Principal component analysis for the transcriptome profiles of three m^6^A modification patterns, showing a remarkable difference on transcriptome between different modification patterns. **e** The proportion of ACRG molecular subtypes in the three modification patterns. MSI subtype, red; EMT subtype, blue; MSS/TP53+ subtype, green; MSS/TP53- subtype, yellow. **f** Functional annotation for m^6^A-related genes using GO enrichment analysis. The color depth of the barplots represented the number of genes enriched

To explore the biological behaviors among these distinct m^6^A modification patterns, we performed GSVA enrichment analysis. As shown in Fig. 2c and Table S5, m6Acluster-A was markedly enriched in stromal and carcinogenic activation pathways such as ECM receptor interaction, TGF beta signaling pathway, cell adhesion and MAPK signaling pathways. m6Acluster-B presented enrichment pathways associated with immune fully activation including the activation of chemokine signaling pathway, cytokine-cytokine receptor interaction, T cell receptor signaling pathway and Toll like receptor signaling pathways (Fig. 2c). While m6Acluster-C was prominently related to immune suppression biological process (Fig. 2d). To our surprise, subsequent analyses of TME cell infiltration indicated m6Acluster-A was remarkably rich in innate immune cell infiltration including natural killer cell, macrophage, eosinophil, mast cell, MDSC, plasmacytoid dendritic cell (Fig. 3a and Table S4). However, patients with this m^6^A modification pattern did not show a matching survival advantage (Fig. 2b). Previous studies demonstrated that tumors with immune-excluded phenotype also showed the presence of abundant immune cells, while these immune cells were retained in the stroma surrounding tumor cell nests rather than penetrate their parenchyma. The activation of stroma in TME were considered T-cell suppressive [39]. The results from GSVA analyses have revealed cluster A modification pattern was significantly associated with stromal activation. Therefore, we speculated that stromal activation in cluster A inhibited the antitumor effect of immune cells. Subsequent analyses showed that stroma activity was significantly enhanced in cluster A such as the activation of epithelial-mesenchymal transition (EMT), transforming growth factor beta (TGFb) and angiogenesis pathways, which confirmed our speculation (Fig. 3b) Based on the above analyses, we were surprised to find three m^6^A modification patterns had significantly distinct TME cell infiltration characterization. Cluster A was classified as immune-excluded phenotype, characterized by innate immune cell infiltration and stromal activation; cluster B was classified as immune-inflamed phenotype, characterized by adaptive immune cell infiltration and immune activation; cluster C was classified as immune-desert phenotype, characterized by the suppression of immunity (Figs. 2c-d and 3a-b). We then used the CIBERSORT method, a deconvolution algorithm using support vector regression for determining the immune cell type in tumors, to compare the component differences of immune cells among the three m^6^A modification patterns. We found that there were no significant differences on the compositions of TME cell types between the three m^6^A modification patterns, which suggested that m^6^A methylation modification did not change TME infiltrating-cell types of tumors (Figure S2K).

We then examined the specific correlation between each TME infiltration cell type and each m^6^A regulator using spearman’s correlation analyses (Figure S3A). We focused on the regulator KIAA1429, a m^6^A methyltransferases, and revealed its significantly negative correlation with numerous TME infiltrating immune cells. We used ESTIMATE algorithm to quantify the overall infiltration of immune cells between high and low KIAA1429 expression patients. The results showed that low expression of KIAA1429 exhibited high immune scores, which meant that the TME with low expression of KIAA1429 existed a significantly increased immune cell infiltration, thus confirming the above findings (Figure S3B). We then explored the specific difference of 23 TME infiltrating immune cells between high and low KIAA1429 expression patients. We found tumors with low expression of KIAA1429 presented significantly increased infiltration in 23 TME immune cells compared to patients with high expression (Figure S3C). Recent studies paid special attention to the mechanism of m^6^A modification regulating the activation of dendritic cells (DCs). DCs, which are responsible for antigen presentation and the activation of naive T cells, are a bridge connecting innate and adaptive immunity, and their activation depending on the high expression level of MHC molecules, costimulatory factors and adhesion factors [40]. Our study indicated that tumors with low expression of KIAA1429 showed significant more enrichment of TME DCs infiltration including activated DCs, immature DCs, and plasmacytoid DCs. We also noted that the decreased expression of KIAA1429 resulted in the comprehensively elevated expression of MHC molecules, costimulatory molecules, and adhesion molecules (Figure S3D). Subsequent pathway enrichment analyses, as expected, tumors with low KIAA1429 expression exhibited an obvious enhancement in immune activation pathways including the pathway of antigen processing and presentation, C-type lectin receptor, NOD-like receptor, T cell receptor, Toll-like receptor and NF-κB signaling pathway (Figure S3E). It was interesting that the immune-related pathway enhancements were accompanied by the increased expression of immunological checkpoint molecules PD1/L1 (Figure S3D-S3E). So we investigated whether the expression of KIAA1429 regulator affected the therapeutic efficacy of immune checkpoint blockade. In anti-PD-L1 immunotherapy cohort (IMvigor210), a survival benefit trend was observed in patients with low expression of KIAA1429 (Figure S3F). In anti-PD-1 immunotherapy cohort (GSE78220), we did not observe a significantly prolonged survival owing to the few samples (Figure S3G). From above, we could speculate that KIAA1429-mediated m^6^A methylation modification may promote the activation of TME DCs, thus enhancing the intratumoral antitumor immune response.

### m^6^A methylation modification patterns in ACRG cohort

To further explore the characteristics of these m^6^A modification phenotypes in the different clinical traits and biological behaviors, we fixed attention on the ACRG cohort, which comprised 300 gastric cancer patients and offered the most comprehensive clinical annotation. Similar to all GC datasets clustering, unsupervised clustering also discovered three fully distinct patterns of m^6^A modification in ACRG cohort (Figure S4A-S4D and Fig. 3c-d). There was significant distinction existed on the m^6^A transcriptional profile among three different m^6^A modification patterns (Fig. 3d). m6Acluster A was characterized by the increased expression of FTO and HNRNPA2B1, and presented variable decreases in other m^6^A regulators; m6Acluster B showed high expression of ELAVL1, HNRNPC, LRPPRC, METTL14, RBM15, RBM15B, YTHDC2 and YTHDF2; and m6Acluster C exhibited significant increases in the expression of FMR1 IGF2BP1, WTAP, ZC3H13 and YTHDF1. Patients with EMT molecular subtypes were characterized by the m6Acluster-A methylation modification patterns, while MSI subtypes were characterized by the m6Acluster-B modification patterns. We also noted that tumors with m6Acluster-A patterns presented poorer differentiation and were enriched in the diffuse histological subtype. A better tumor differentiation was observed in the m6Acluster-B and m6Acluster-C patterns, which were enriched in the intestinal histological subtype. In gastric cancer, the EMT molecular subtype and diffuse histological type was markedly linked to a poorer survival, while MSI linked to a better clinical outcome. Therefore, the tumors characterized by m6Acluster-A modification patterns were significantly correlated with stromal activation, high malignancy and rapid progression (Fig. 3c). One-way ANOVA test also confirmed the remarkable differences on m^6^A regulator expression between three key m^6^A modification patterns. Prognostic analysis also revealed m6Acluster B to be markedly related to prolonged survival, while m6Acluster A and m6Acluste C were characterized by poorer survival (Figure S4E-S4F). Consistent with the above findings, most patients with EMT subtypes were clustered into m6Acluster A and almost no EMT subtypes were in m6Acluster B, which confirmed again that m6Acluster A was significantly relevant to the stromal activation and m6Aclustre B relevant to the immune activation (Fig. 3e and Table S6).

### Generation of m^6^A gene signatures and functional annotation

**Fig. 4:**
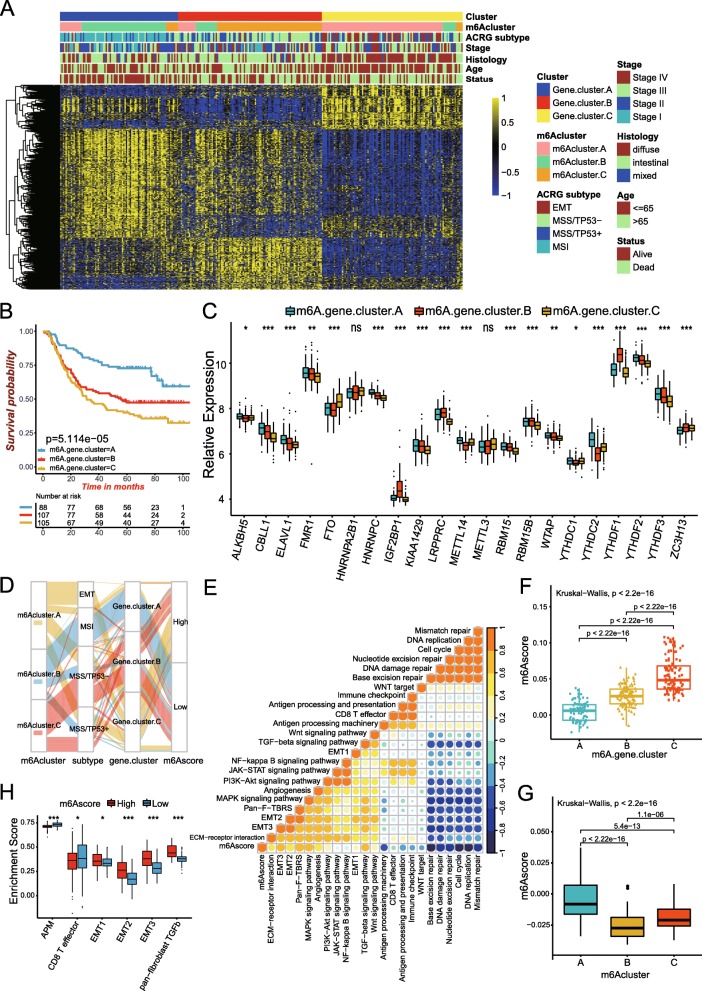
Construction of m^6^A signatures. **a** Unsupervised clustering of overlapping m^6^A phenotype-related genes in ACRG cohorts to classify patients into different genomic subtypes, termed as m^6^A gene cluster A-C, respectively. The gene clusters, m6Aclusters, ACRG molecular subtypes, tumor stage, histology, survival status and age were used as patient annotations. **b** Kaplan-Meier curves indicated m^6^A modification genomic phenotypes were markedly related to overall survival of 300 patients in ACRG cohort, of which 88 cases were in gene cluster A, 107 cases in gene cluster B and 105 cases in gene cluster C (*P* < 0.0001, Log-rank test). **c** The expression of 21 m^6^A regulators in three gene cluster. The upper and lower ends of the boxes represented interquartile range of values. The lines in the boxes represented median value, and black dots showed outliers. The asterisks represented the statistical *p* value (*P < 0.05; **P < 0.01; ***P < 0.001). The one-way ANOVA test was used to test the statistical differences among three gene clusters. **d** Alluvial diagram showing the changes of m6Aclusters, ACRG molecular subtypes, gene cluster and m6Ascore. **e** Correlations between m6Ascore and the known gene signatures in ACRG cohort using Spearman analysis. Negative correlation was marked with blue and positive correlation with orange. **f** Differences in m6Ascore among three gene clusters in ACRG cohort. The Kruskal-Wallis test was used to compare the statistical difference between three gene clusters (*P* < 0.001). **g** Differences in m6Ascore among three m^6^A modification patterns in ACRG cohort (*P* < 0.001, Kruskal-Wallis test). **h** Differences in stroma-activated pathways between high m6Ascore and low m6Ascore groups. APM, antigen processing machinery; EMT, epithelial-mesenchymal transition; TGFb, transforming growth factor beta. The upper and lower ends of the boxes represented interquartile range of values. The lines in the boxes represented median value. The asterisks represented the statistical *p* value (*P < 0.05; **P < 0.01; ***P < 0.001)

To further investigate the potential biological behavior of each m^6^A modification pattern, we determined 718 m^6^A phenotype-related DEGs using limma package (Figure S4G and Table S7). The clusterProfiler package was used to perform GO enrichment analysis for the DEGs. The biological processes with significant enrichment were summarized in Table S8. Surprisingly, these genes showed enrichment of biological processes remarkably related to m^6^A modification and immunity, which confirmed again that m^6^A modification played a non-negligible role in the immune regulation in tumor microenvironment (Fig. 3f). To further validate this regulation mechanism, we then performed unsupervised clustering analyses based on the obtained 718 m^6^A phenotype-related genes in order to classify patients into different genomic subtypes. Consistent with the clustering grouping of m^6^A modification patterns, the unsupervised clustering algorithm also revealed three distinct m^6^A modification genomic phenotypes and we named these three clusters as m^6^A gene cluster A-C, respectively (Figure S5A-S5D, Fig. 4a and Table S6). This demonstrated that three distinct m^6^A methylation modification patterns did exist in gastric cancer. We observed that tumors in m^6^A gene cluster C patterns also exhibited poorer differentiation and were enriched in the diffuse histological subtype. The opposite patterns were observed in m^6^A gene cluster A and cluster B. Patients with alive status or MSI subtypes were mainly concentrated in the m^6^A gene cluster A, while patients with clinical stage IV or EMT molecular subtypes were characterized by the m^6^A gene cluster C patterns (Fig. 4a). Analysis also indicated three distinct gene clusters were characterized by different signature genes (Fig. 4a). Eighty-eight of three hundred patients with gastric cancer were clustered in gene cluster A, which were proved to be related to better prognosis. While patients in gene cluster C (105 patients) experienced the outcome of poorer prognosis. An intermediate prognosis was observed in gene cluster C, with 107 patients clustered (Fig. 4b). In the three m^6^A gene clusters, the prominent differences in the expression of m^6^A regulators were observed, which was in accordance with the expected results of m^6^A methylation modification patterns (Fig. 4c).

### Characteristics of clinical and transcriptome traits in m^6^A-related phenotypes

To reveal the role of m^6^A-related phenotypes in the TME immune regulation, we studied the expression of chemokine and cytokine characterizing three gene clusters. The selected cytokine and chemokine were extracted from published literature, of which, TGRB1, SMAD9, TWIST1, CLDN3, TGFBR2, ACTA2, COL4A1, ZEB1 and VIM were considered to be associated with the transcripts of transforming growth factor (TGF)b/EMT pathway. PD-L1, CTLA-4, IDO1, LAG3, HAVCR2, PD-1, PD-L2, CD80, CD86, TIGIT and TNFRSF9 were considered to be related to the transcripts of immune checkpoints. TNF, IFNG, TBX2, GZMB, CD8A, PRF1, GZMA, CXCL9 and CXCL10 were to be correlated with the transcripts of immune activation [29, 32]. We found the mRNAs relevant to TGFb/EMT pathway were significantly upregulated in gene cluster C, which demonstrated that this cluster was deemed as stromal-activated group. While gene cluster A showed high expression of mRNAs related to immune activation transcripts. This suggested that gene cluster A could be classified as the immune-activation group (Figure S5F-S5H). To better depict the function of m^6^A signature genes, we examined the known signatures in patients with gastric cancer (Figure S5E). The results also confirmed that gene cluster C was characterized by the status of stromal activation and cancer promotion, and gene cluster A was significantly related to immune activation status (Figure S5E-S5H). Consistent with the above findings, as shown in Fig. 4d and Table S6, almost all (41 of 45, 91%) patients with EMT subtype (molecular subtypes in ACRG cohort) were classified into gene cluster C, which was relevant to the worse survival outcome.

**Fig. 5:**
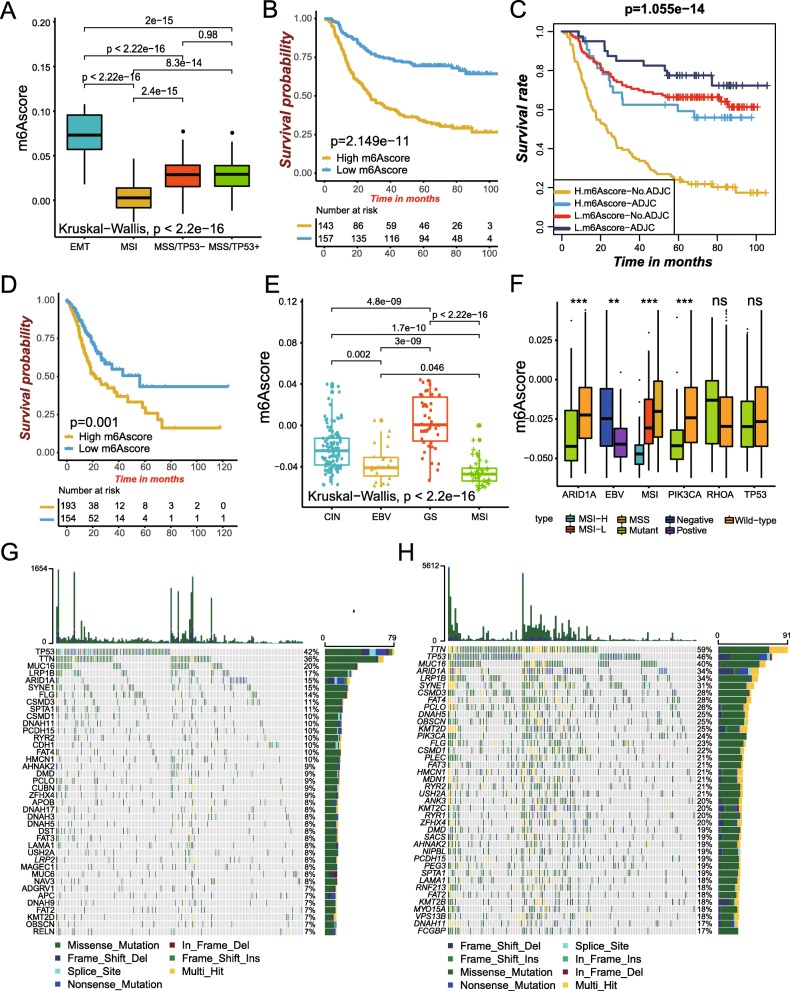
Characteristics of m^6^A modification in TCGA molecular subtypes and tumor somatic mutation. **a** Differences in m6Ascore between different ACRG molecular subtypes. The Kruskal-Wallis test was used to compare the statistical difference between four ACRG molecular subtypes (*p* < 0.0001). **b** Survival analyses for low (157 cases) and high (143 cases) m6Ascore patient groups in ACRG cohort using Kaplan-Meier curves (HR, 3.0 (2.12–4.21); *P* < 0.0001, Log-rank test). **c** Survival analyses for subgroup patients stratified by both m6Ascore and treatment with adjuvant chemotherapy using Kaplan-Meier curves. H, high; L, Low; ADJC, adjuvant chemotherap (*P* < 0.0001, Log-rank test). **d** Survival analyses for low (157 cases) and high (143 cases) m6Ascore patient groups in the TCGA-STAD cohort using Kaplan-Meier curves (HR, 1.81(1.26–2.62); *P* = 0.001, Log-rank test). **e** Differences in m6Ascore between different TCGA-STAD molecular subtypes. The upper and lower ends of the boxes represented interquartile range of values. The lines in the boxes represented median value. The Kruskal-Wallis test was used to compare the statistical difference between four TCGA-STAD molecular subtypes (*p* < 0.0001). GS, genome stable; MSI, microsatellite instability; EBV, EBV infection; CIN, chromosomal instability. **f** Differences in m6Ascore among different of microsatellite subtypes. The upper and lower ends of the boxes represented interquartile range of values. The lines in the boxes represented median value. The asterisks represented the statistical *p* value (*P < 0.05; **P < 0.01; ***P < 0.001). MSS, microsatellite stable; MSI-H, high microsatellite instability; MSI-L, low microsatellite instability. **g**, **h** The waterfall plot of tumor somatic mutation established by those with high m6Ascore (**g**) and low m6Ascore (**h**). Each column represented individual patients. The upper barplot showed TMB, The number on the right indicated the mutation frequency in each gene. The right barplot showed the proportion of each variant type

The above results showed again that m^6^A methylation modification played a non-negligible regulation role in shaping different TME landscapes. However, these analyses were only based on the patient population and could not accurately predict the pattern of m^6^A methylation modification in individual patients. Considering the individual heterogeneity and complexity of m^6^A modification, based on these phenotype-related genes, we constructed a set of scoring system to quantify the m^6^A modification pattern of individual patients with gastric cancer, we termed as m6Ascore. The alluvial diagram was used to visualize the attribute changes of individual patients (Fig. 4d). To better illustrate the characteristics of m^6^A signature, we also tested the correlation between the known signatures and the m6Ascore (Fig. 4e and Table S9). Kruskal-Wallis test revealed significant difference on m6Ascore between m^6^A gene clusters. Gene cluster A showed the lowest median score while gene cluster C had the highest median score, which indicated that low m6Ascore could be closely linked to immune activation-related signatures, whereas high m6Ascore could be linked to stromal activation-related signatures (Fig. 4f). More importantly, m6Acluster A showed the significantly increased m6Ascore compared to the other clusters and m6Acluster B presented the lowest median score (Fig. 4g). The analyses for the activity of stroma-related pathways indicated high scores were significantly associated with enhanced activation of stromal pathways (Fig. 4h). In addition, patients with EMT subtypes also showed the lowest m6Ascore compared to other three ACRG molecular subtypes (Fig. 5a). The above results strongly suggested that low m6Ascore was significantly correlated with immune-activation and high m6Ascore was correlated with stromal-activation. The m6Ascore could better evaluate the m^6^A modification patterns of individual tumor, and further evaluate tumors’ TME cell-infiltration characterization, in order to distinguish the true and false nature of TME immune infiltration.

Next, we sought to further identify the value of m6Ascore in predicting patients’ outcome. With the cutoff value 0.0291 determined by survminer package, patients were divided into low or high m6Ascore group. Patients with low m6Ascore demonstrated a prominent survival benefit (HR 3.0 (2.12–4.21); Fig. 5b), with 5-year survival rate twice than patients with high m6Ascore (69.4% vs 33.5%). We tested whether the m6Ascore could serve as an independent prognostic biomarker for gastric cancer. Multivariate Cox regression model analysis, which included the factors of patients’ age, gender, TNM status, histological type, MSI status, TP53 status and ACRG molecular subtypes, confirmed m6Ascore as a robust and independent prognostic biomarker for evaluating patient outcomes (HR 2.54(1.71–3.8); Figure S6A). We specifically examined the ability of m6Ascore signature to predict the efficacy of adjuvant chemotherapy in patients with gastric cancer. We found that patients with low m6Ascore showed significant therapeutic advantages among patients who also received adjuvant chemotherapy, with 5-year survival rate 77.5% vs 59.2% (Fig. 5c). Another results obtained indicated that the prediction power of m6Ascore was not interfered by adjuvant chemotherapy, and both in patients receiving chemotherapy or not, low m6Ascore group always showed the obvious survival advantage (Fig. 5c). In addition, we revealed that younger patients, diffuse histological subtype and advanced patients were significantly associated with a higher m6Ascore, which meant that these patients were characterized by the m6Acluster-A modification patterns and immune-excluded phenotype, with a poorer clinical outcome. These results demonstrated m6Ascore could be also used to evaluate certain clinical characteristics of patients such as MSI status, molecular subtypes, histological subtypes as well as clinical stage, etc. (Figure S6B).

### Characteristics of m^6^A modification in TCGA molecular subtypes and tumor somatic mutation

A comprehensive molecular landscape has been constructed for gastric cancer by TCGA project, which classified gastric cancer into four molecular subtypes including genome stable (GS), microsatellite instability (MSI), EBV infection, and chromosomal instability (CIN). We evaluated the difference of m6Ascore between these molecular subtypes. The higher m6Ascore was obviously concentrated on GS subtype and showed a worse survival in patients, while the lower m6Ascore was concentrated on the subtypes of MSI and EBV infection, which was related to better survival (5-year survival rate, 25.9% vs 43.3%; HR 1.81(1.26–2.62); Fig. 5d-e). The highly microsatellite instability subtype, characterized by better prognosis, was significantly correlated with lower m6Ascore, whereas MSI-Low and MSS had a higher m6Ascore (Fig. 5f). Multivariate analysis for TCGA-STAD cohort also confirmed that m6Ascore could act as an independent prognostic biomarker in gastric cancer (Figure S6C). Previous studies indicated that patients with EBV-positive gastric cancer have been shown to respond to anti-PD-1/L1 antibodies in several studies in spite of the lower MSI or tumor mutation burden (TMB) [41, 42]. In our study, EBV infected patients were markedly associated with lower m6Ascore than CIN and GS subtypes as well as EBV non-infected patients, which implied m6Ascore signature could be a more effective biomarker for the prediction of immunotherapeutic efficacy than MSI and TMB in patients with gastric cancer (Fig. 5e-f). Further research showed that tumors with MSI subtype were mainly characterized by the m6Acluster-B methylation modification patterns, while tumors with MSS subtype were characterized by the m6Acluster-C modification patterns (Figure S7A). The m^6^A regulators ALKBH5, CBLL1, ELAVL1, FMR1, HNRNPC, KIAA1429, METTL14, RBM15, RBM15B, WTAP, YTHDC1, YTHDC2, YTHDF2 and YTHDF3 were significantly up-regulated in MSI subtypes compared to MSS subtypes, while IGF2BP1, YTHDF1 and ZC3H13 were markedly down-regulated (Figure S7B). For EB virus infection, patients with EBV-positive were mainly characterized by the m6Acluster-A methylation modification patterns, while EBV-negative patients did not show a characteristic pattern of m^6^A methylation modification. There were no significant difference on m^6^A modification patterns between EBV-negative patients (Figure S7C). In addition, we found the m^6^A regulators IGF2BP1, KIAA1429, LRPPRC, YTHDF3 and ZC3H13 were remarkably down-regulated in EBV-negative patients than EBV positive patients, while FTO was significantly down-regulated (Figure S7D). The above results suggested that the potential mechanisms on the change of m^6^A modification patterns mediated by EBV and MSI etc. may be that these factors changed the status of m^6^A regulators. These findings could contribute to enhancing our understanding of the mechanisms of the formation of m^6^A modification pattern differences in tumors.

Then, we analyzed the distribution differences of somatic mutation between low and high m6Ascore in TCGA-STAD cohort using maftools package. As shown in Fig. 5g-h, low m6Ascore group presented more extensive tumor mutation burden than the high m6Ascore group, with the rate of the 10th most significant mutated gene 25% versus 10%. The TMB quantification analyses confirmed the low m6Ascore tumors was markedly correlated with a higher TMB (Figure S7E). The m6Ascore and TMB also exhibited a significant negative correlation (Figure S7F). Accumulated evidence demonstrated patients with high TMB status presented a durable clinical response to anti-PD-1/PD-L1 immunotherapy. Therefore, the above results indirectly demonstrated that the difference in tumor m^6^A modification patterns could a crucial factor that mediated the clinical response to anti-PD-1/PD-L1 immunotherapy. And the values of m6Ascore in predicting immunotherapeutic outcomes were also indirectly confirmed.

The clinical trials as well as preclinical researches have revealed patients with higher somatic TMB were correlated with enhanced response, long-term survival and durable clinical benefit when treated with immune checkpoint blockade therapy. The individual altered genes could mediate resistance or sensitivity to immunotherapy. For specific altered genes in TCGA-TSAD such as ARID1A and PIK3CA, mutant type had significantly lower m6Ascore compared to wild type, whereas there was no significant difference in m6Ascore between wild and mutant types in TP53 and RHOA (Fig. 5f). These results would provide novel perspective for exploring the mechanisms of m^6^A methylation modification in the tumor somatic mutations, shaping of TME landing, and roles in immune checkpoint blockade therapy.

### m6A modification patterns in the role of anti-PD-1/L1 immunotherapy

In order to further test the stability of m6Ascore model, we applied m6Ascore signature established in ACRG cohort to other independent gastric cancer cohorts to verify its prognostic value (GSE84437, HR 1.89(1.44–2.49); GSE15459, HR 2.05(1.30–3.22); GSE34942, HR 1.52(0.64–3.63); Figure S8A-S8C). The combined set of all GEO cohorts was validated (HR 1.94(1.62–2.31); Figure S8D). The ability of m6Ascore to predict relapse-free survival was also evaluated (GSE26253, HR 1.33(0.98–1.80); GSE62254, HR 2.53(1.75–3.65); Figure S8E-S8F). Next, we continued to extend the m6Ascore signature to all digestive system tumors including cholangiocarcinoma, colon adenocarcinoma, pancreatic adenocarcinoma, esophageal carcinoma and liver hepatocellular carcinoma (HR 1.4(1.17–1.68); Figure S8G). These data indicated m^6^A modification patterns correlated with better clinical benefit. The predictive advantage evaluated with ROC curves was especially reflected in elderly patients (Figure S8H-S8I).

**Fig. 6:**
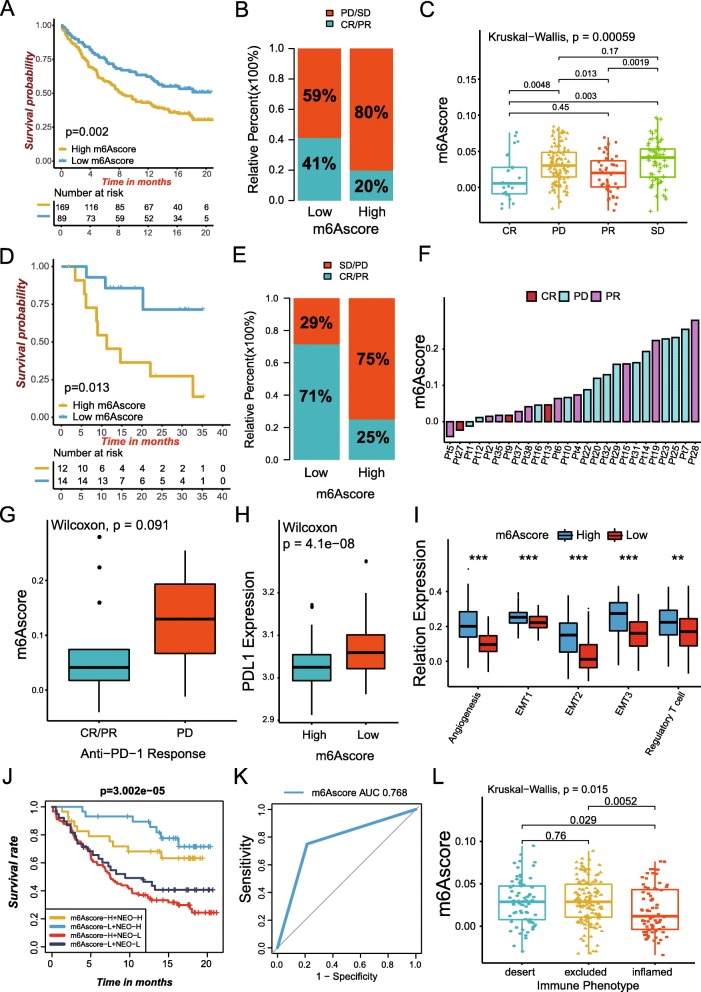
m^6^A modification patterns in the role of anti-PD-1/L1 immunotherapy. **a** Survival analyses for low (89 cases) and high (169 cases) m6Ascore patient groups in the anti-PD-L1 immunotherapy cohort using Kaplan-Meier curves (IMvigor210 cohort; HR, 1.73(1.20–2.48); *P* = 0.002, Log-rank test). **b** The proportion of patients with response to PD-L1 blockade immunotherapy in low or high m6Ascore groups. SD, stable disease; PD, progressive disease; CR, complete response; PR, partial response. Responser/Nonresponer: 41%/58% in the low m6Ascore groups and 20%/80% in the high m6Ascore groups. **c** Distribution of m6Ascore in distinct anti-PD-L1 clinical response groups. **d** Survival analyses for low and high m6Ascore patient groups in the anti-PD1 immunotherapy cohort using Kaplan-Meier curves (GSE78220 cohort; HR, 4.58(1.23–17.10); *P* = 0.013, Log-rank test). **e** The proportion of patients with response to PD-1 blockade immunotherapy in low or high m6Ascore groups. Responser/Nonresponer: 71%/29% in the low m6Ascore groups and 25%/75% in the high m6Ascore groups. **f** The correlation of m6Ascore with clinical response to anti-PD-1 immunotherapy. Pt, patients. PD, blue; PR, purple; CR, red. **g** Differences in m6Ascore among distinct anti-PD-1 clinical response groups. **h** Differences in PD-L1 expression between low and high m6Ascore groups (*p* < 0.0001, Wilcoxon test). **i** Differences in stroma-activated pathways and abundance of regulatory T cells (considered as immune suppression) between low m6Ascore and high m6Ascore groups in anti-PD-L1 immunotherpy cohort (*P < 0.05; **P < 0.01; ***P < 0.001). **j** Survival analyses for patients receiving anti-PD-L1 immunotherapy stratified by both m6Ascore and neoantigen burden using Kaplan-Meier curves. H, high; L, Low; NEO, neoantigen burden (*P* < 0.0001, Log-rank test). **k** The predictive value of the quantification of m^6^A modification patterns in patients treated with anti-PD-1/L1 immunotherapy (AUC, 0.768). **l** Differences in m6Ascore among distinct tumor immune phenotypes in IMvigor210 cohort. The lines in the boxes represented median value (*p* = 0.015, Kruskal-Wallis test)

Immunotherapies represented by PD-L1 and PD-1 blockade has undoubtedly emerged a major breakthrough in cancer therapy. We investigated whether the m^6^A modification signature could predict patients’ response to immune checkpoint blockade therapy based on two immunotherapy cohorts. In both anti-PD-L1 cohort (IMvigor210) and anti-PD-1 cohort (GSE78220), patients with low m6Ascore exhibited significantly clinical benefits and a markedly prolonged survival (Fig. 6a-g; IMvigor210, HR 1.73(1.20–2.48), Fig. 6a; GSE78220, HR 4.58(1.23–17.10), Fig. 6d). The significant therapeutic advantages and clinical response to anti-PD-1/L1 immunotherapy in patients with low m6Ascore compared to those with high m6Ascore were confirmed (Fig. 6b-c and e-g). In addition, patients with low m6Ascore showed a obviously high expression of PD-L1, which indicated a potential response to anti-PD-1/L1 immunotherapy (Fig. 6h). Further research revealed that regulatory T-cells and TME stroma were significantly activated in tumors with high m6Ascore, which mediated immune tolerance of tumors (Fig. 6i). Tumor neoantigen burden, closely linked to immunotherapeutic efficacy, was also assessed. We found patients with combination of low m6Ascore and high neoantigen burden showed a great survival advantage (Fig. 6j). The above implied that the quantification of m^6^A modification patterns was a potential and robust biomarker for prognosis and clinical response assessment of immunotherapy (Fig. 6k). The immune phenotypes of tumors in the IMvigor210 cohort has been detected, so we investigated the difference of m6Ascore among different phenotypes. We found that higher m6Ascore was remarkably associated with exclusion and desert immune phenotypes, and checkpoint inhibitors were difficult to exert antitumor effect in these phenotype (Fig. 6l). In summary, our work strongly indicated that m^6^A methylation modification patterns was significantly correlated with tumor immune phenotypes and response to anti-PD-1/L1 immunotherapy, and the established m^6^A modification signature would contribute to predicting the response to anti-PD-1/L1 immunotherapy.

## Discussion

Increasing evidence demonstrated that m^6^A modification took on an indispensable role in inflammation, innate immunity as well as antitumor effect through interaction with various m^6^A regulators. As most studies focus on single TME cell type or single regulator, the overall TME infiltration characterizations mediated by integrated roles of multiple m^6^A regulators are not comprehensively recognized. Identifying the role of distinct m^6^A modification patterns in the TME cell infiltration will contribute to enhancing our understanding of TME antitumor immune response, and guiding more effective immunotherapy strategies.

Here, based on 21 m^6^A regulators, we revealed three distinct m^6^A methylation modification patterns. These three patterns had significantly distinct TME cell infiltration characterization. Cluster A was characterized by the activation of innate immunity and stroma, corresponding to immune-excluded phenotype; cluster B was characterized by the activation of adaptive immunity, corresponding to immune-inflamed phenotype; cluster C was characterized by the suppression of immunity, corresponding to immune-desert phenotype. The immune-excluded and immune-desert phenotypes could be regarded as non-inflamed tumors. The immune-inflamed phenotype, known as hot tumor, show by a large number of immune cell infiltration in TME [39, 43, 44]. Although the immune-excluded phenotype also showed the presence of abundant immune cells, the immune cells were retained in the stroma surrounding tumor cell nests rather than penetrate their parenchyma. The stroma could be confined to the tumor envelope or may penetrate the tumor itself, making the immune cells appear to be really inside the tumor [45–47]. The immune-desert phenotypes were associated with immune tolerance and ignorance, and lack of activated and priming T-cell [48]. Consistent with the above definitions, we found cluster A exhibited a significant stroma activation status, including the highly expressed angiogenesis, EMT and TGF-β pathways, which were considered T-cell suppressive. Combined with the TME cell-infiltrating characteristics in each cluster, it confirmed the reliability of our classification of immune phenotypes for different m^6^A modification patterns. Therefore, after fully exploring the TME cell–infiltrating characterization induced by distinct m^6^A modification patterns, it was not surprising that cluster A had the activated innate immunity but poorer prognosis.

Further, in this study, the mRNA transcriptome differences between distinct m^6^A modification patterns have been proved to be significantly associated with m^6^A and immune related biological pathways. These differentially expressed genes were considered as m^6^A-related signature genes. Similar to the clustering results of the m^6^A modification phenotypes, three genomic subtypes were identified based on m^6^A signature genes, which were also significantly correlated with stromal and immune activation. This demonstrated again that the m^6^A modification was of great significance in shaping different TME landscapes. Therefore, a comprehensive assessment of the m^6^A modification patterns will enhance our understanding of TME cell-infiltrating characterization. Considering the individual heterogeneity of m^6^A modification, it was urgently demanded to quantify the m^6^A modification patterns of individual tumor. For that, we established a set of scoring system to evaluate the m^6^A modification pattern of individual patients with gastric cancer—the m^6^A gene signature. The m^6^A modification pattern characterized by immune-excluded phenotype exhibited a higher m6Ascore, while the pattern characterized by immune-inflamed phenotype showed a lower m6Ascore. In addition, In IMvigor210 cohort with the determined immune phenotype, these results were well validated [33]. This suggested m6Ascore was a reliable and robust tool for comprehensive assessment of individual tumor m^6^A modification patterns, which could be used to further determine the TME infiltration patterns, that was, tumor immune phenotypes. Integrated analyses also demonstrated that m6Ascore was an independent prognostic biomarker in gastric cancer. Patients with EBV and MSI subtypes, sensitive to checkpoint immunotherapy [42], was significantly related to lower m6Ascore. Considering the low mutation burden but high immune infiltration in EBVþ tumors [41], our m6Ascore showed a predictive advantage in precision immunotherapy for gastric cancer.

Our data also revealed a markedly negative correlation between m6Ascore and tumor mutation burden. Consistent with previous studies, EMT and GS molecular subtypes demonstrated the lowest m6Ascore, underlining the core role of stromal activation in resistance to checkpoint immunotherapy [49, 50]. This indicated that response to checkpoint immunotherapy was not only associated with antigen processing, and improved cytolytic activity, but also related to suppression of angiogenesis, fibroblast activation, TGF beta pathway components and the EMT. Previous studies confirmed that the EMT- and TGFbeta-related pathway activation resulted in decreased trafficking of T-cell into tumors as well as their weakened tumor killing effects [33, 49]. The above suggested that the activated stromal TME in the activated immune TME could mediate therapeutic resistance to immune-checkpoint blockade, as well as influence the individual precise immunotherapy of gastric cancer. In this work, we showed m^6^A methylation modification patterns played a nonnegligible role in shaping different stromal and immune TME landscape, implying m^6^A modification could affect the therapeutic efficacy of immune checkpoint blockade. The m^6^A gene signature with integrated various biomarkers including mutation load, neoantigen load, PD-L1 expression, stromal and immune TME and MSI status, could be the more effective predictive strategy for immunotherapy. We also confirmed the predictive value of the m6Ascore in two cohort with anti-PD-1 and anti-PD-L1 immunotherapy. A significantly difference on m6Ascores existed between non-responders and responders.

In short, in clinical practice, the m6Ascore could be used to comprehensively evaluate the m^6^A methylation modification patterns as well as their corresponding TME cell infiltration characterization within individual patient, further to determine the immune phenotypes of tumors and guide the more effective clinical practice. We also demonstrated the m6Ascore could be utilized for assessing patients’ clinicopathological features including stages of tumor inflammation, tumor differentiation levels, clinical stages, histological subtypes, molecular subtypes, genetic variation, MSI status, EBV infection and tumor mutation burden etc. The detailed relationships between m6Ascore and clinicopathological features could be found in our study. Similarly, m6Ascore could act as an independent prognostic biomarker for predicting patients’ survival. We could also predict the efficacy of adjuvant chemotherapy and the patients’ clinical response to anti-PD-1/PD-L1 immunotherapy through m6Ascore. More importantly, this study has yielded several novel insights for cancer immunotherapy that targeting m^6^A regulators or m^6^A phenotype-related genes for changing the m^6^A modification patterns, and further reversing the adverse TME cell infiltration characterization, that was the transformation of “cold tumors” into “hot tumors”, may contribute to exploiting the development of novel drug combination strategies or novel immunotherapeutic agents in the future. Our findings provided novel ideas for improving the patients’ clinical response to immunotherapy, identifying different tumor immune phenotypes and promoting personalized cancer immunotherapy in the future.

## Conclusions

In conclusion, this work demonstrated the extensive regulation mechanisms of m^6^A methylation modification on tumor microenvironment. The difference of m^6^A modification patterns was a factor that could not be ignored to cause the heterogeneity and complexity of individual tumor microenvironment. The comprehensive evaluation of individual tumor m^6^A modification pattern will contribute to enhancing our understanding of tumor microenvironment cell-infiltrating characterization and guiding more effective immunotherapy strategies.

## Supplementary information

**Additional file 1:Figure S1.** Overview of study design and prognostic analysis of 21 m^6^A regulators. **Figure S2.** Correlation between writer gene expression/mutation and eraser gene expression. **Figure S3.** Correlation between TME infiltration cells and m^6^A regulators and the roles of KIAA1429 in activation of dendritic cells. **Figure S4.** Unsupervised clustering of 21 m^6^A regulators in the ACRG gastric cancer cohort. **Figure S5.** Characteristics of cytokine transcriptome, chemokine transcriptome and known signatures in distinct gene clusters. **Figure S6.** The prognostic value of m6Ascore and correlation between the clinicopathological features and m6Ascore. **Figure S7.** The effect of microsatellite status and EB virus infection on the three m^6^A modification patterns and m6A regulators. **Figure S8.** Prognostic value of m6Ascore in gastric cancer cohorts and digestive cancer cohorts.

**Additional file 2:Table S1.** Basic information of datasets included in this study for identifying distinct m^6^A methylation modification patterns. **Table S2.** The gene sets used in this work for marking each TME infiltration cell type. **Table S3.** Spearman correlation analysis of the 21 m^6^A modification regulators. **Table S4.** Estimating relative abundance of tumor microenvironment cells in 1059 gastric cancer patients by the Single-Sample Gene-Set. **Table S5.** The activation states of biological pathways in distinct m^6^A modification patterns by GSVA enrichment analysis. **Table S6.** The changes of m6Aclusters, ACRG molecular subtypes, gene clusters and m6Ascore. **Table S7.** Prognostic analysis of 718 m^6^A phenotype-related genes using a univariate Cox regression model. **Table S8.** Functional annotation for m^6^A phenotype -related genes (Gene Ontology-Biological process). **Table S9.** Spearman correlation between m6Ascore and other known signatures within the gastric cancer.

## Data Availability

All data used in this work can be acquired from the Gene-Expression Omnibus (GEO; https://www.ncbi.nlm.nih.gov/geo/) and the GDC portal (https://portal.gdc.cancer.gov/).

## Abbreviations

CIN: Chromosomal instability
CNV: Copy number variation
DCs: Dendritic cells
DEGs: Differentially expressed genes
EMT: Epithelial-mesenchymal transition
GEO: Gene-Expression Omnibus
GS: Genome stable
GSVA: Gene set variation analysis
HR: Hazard ratios
ICB: Immunological checkpoint blockade
m^6^A: N6-methyladenosine
MSI: Microsatellite instability
Pan-F-TBRS: Pan-fibroblast TGFb response signature
PCA: Principal component analysis
ssGSEA: Single-sample gene-set enrichment analysis
TCGA: The Cancer Genome Atlas
TGFb: Transforming growth factor beta
TME: tumor microenvironment
