# Supplementary material for: m6A regulator-mediated methylation modification patterns and tumor microenvironment infiltration characterization in gastric cancer
Source: Mol Cancer. 2020 Mar 12;19:53. doi: 10.1186/s12943-020-01170-0 (PMC7066851; doi:10.1186/s12943-020-01170-0)
Supplement: Supplementary file 1 — Additional file 1:Figure S1. Overview of study design and prognostic analysis of 21 m6A regulators. Figure S2. Correlation between writer gene expression/mutation and eraser gene expression. Figure S3. Correlation between TME infiltration cells and m6A regulators and the roles of KIAA1429 in activation of dendritic cells. Figure S4. Unsupervised clustering of 21 m6A regulators in the ACRG gastric cancer cohort. Figure S5. Characteristics of cytokine transcriptome, chemokine transcriptome and known signatures in distinct gene clusters. Figure S6. The prognostic value of m6Ascore and correlation between the clinicopathological features and m6Ascore. Figure S7. The effect of microsatellite status and EB virus infection on the three m6A modification patterns and m6A regulators. Figure S8. Prognostic value of m6Ascore in gastric cancer cohorts and digestive cancer cohorts. [file 12943_2020_1170_MOESM1_ESM.pdf]

Figure S1. Overview of study design and prognostic analysis of 21 m⁶A regulators.

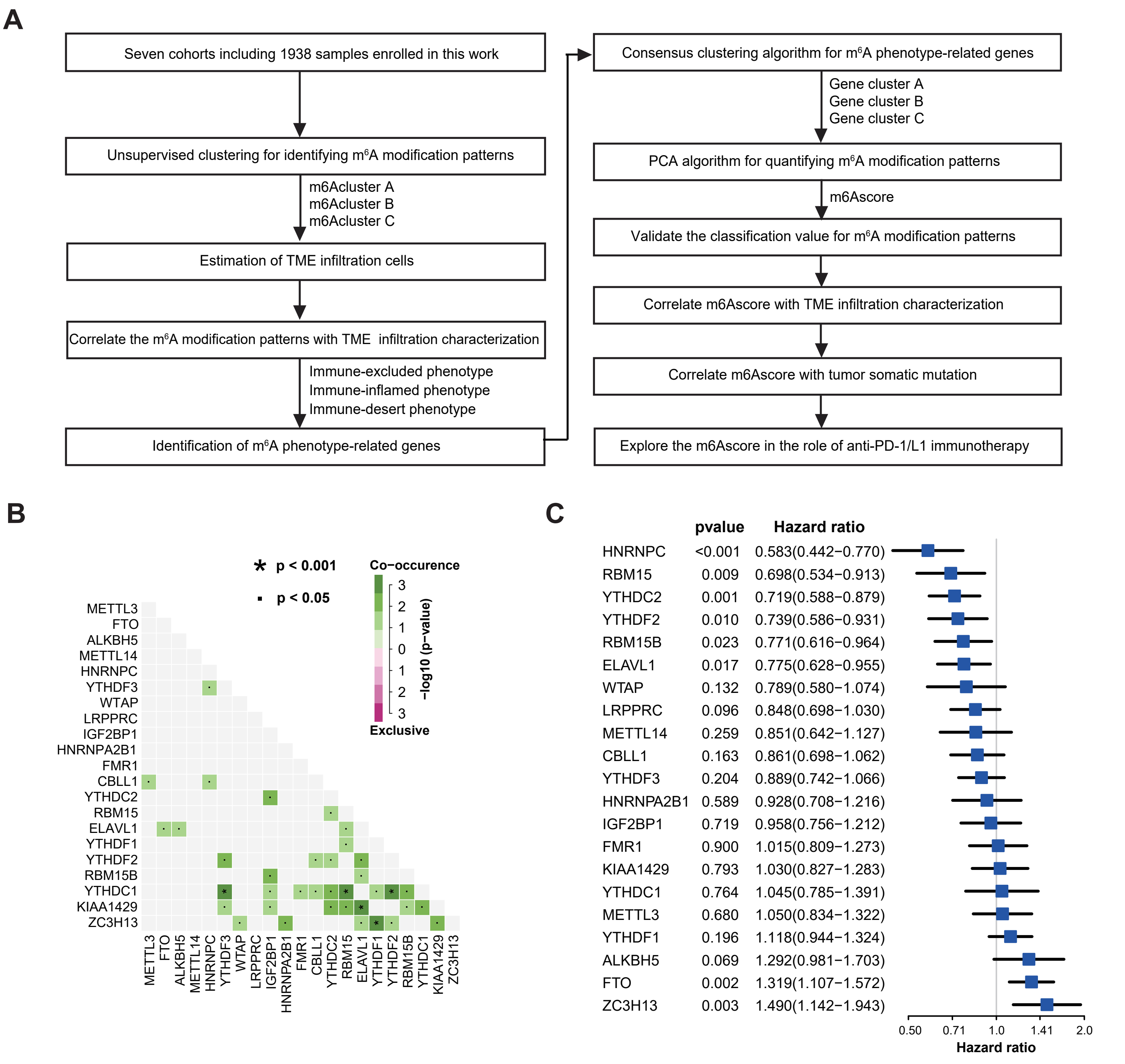

(A) Overview of this work. **(B)** The mutation co-occurrence and exclusion analyses for 21 m⁶A regulators. Co-occurrence, green; Exclusion, purple. **(C)** The prognostic analyses for 21 m⁶A regulators in the five gastric cancer cohorts using a univariate Cox regression model. Hazard ratio >1 represented risk factors for survival and hazard ratio <1 represented protective factors for survival.

Figure S2. Correlation between writer gene expression/mutation and eraser gene expression.

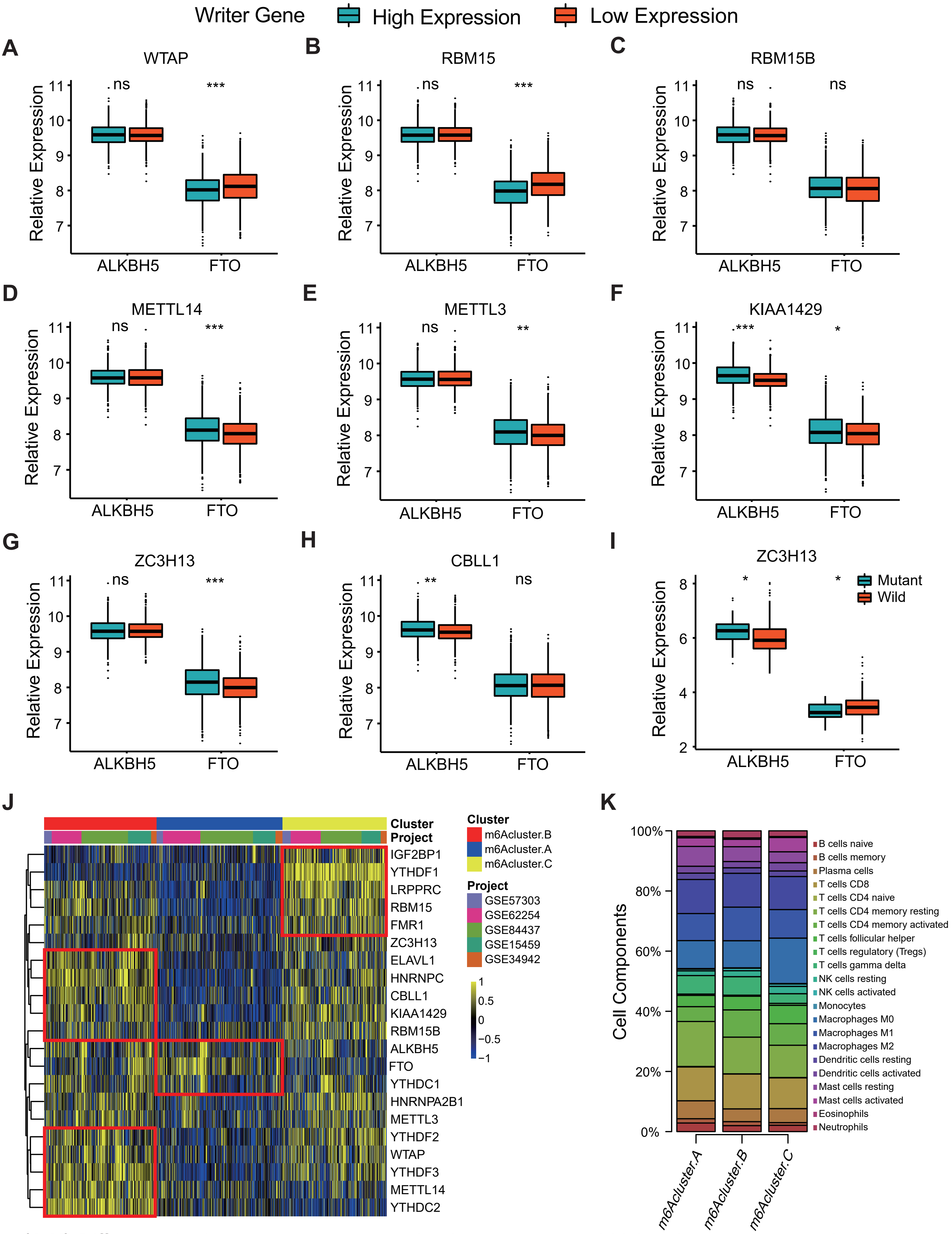

(A-H) Difference in the eraser gene expression between low and high writer gene expression groups. Writer gene, WTAP, RBM15, RBM15B, METTL14, METTL3, KIAA1429, CBLL1, ZC3H1; Eraser gene, ALKBH5, FTO. The upper and lower ends of the boxes represented interquartile range of values. The lines in the boxes represented median value, and black dots showed outliers. The asterisks represented the statistical p value.(*P < 0.05; **P < 0.01; ***P < 0.001) **(I)** Difference in the eraser gene expression between ZC3H13-mutant and wild types. **(J)** Unsupervised clustering of 21 m^{6}A regulators in the five independent gastric cancer cohorts. The m6Aclusters and cohort names were used as patient annotations. Each column represented patients and each row represented m^{6}A regulators. **(K)** The component differences of immune cells among the three m^{6}A modification patterns analyzed by CIBERSORT.

Figure S3. Correlation between TME infiltration cells and m⁶A regulators and the roles of KIAA1429 in activation of dendritic cells.

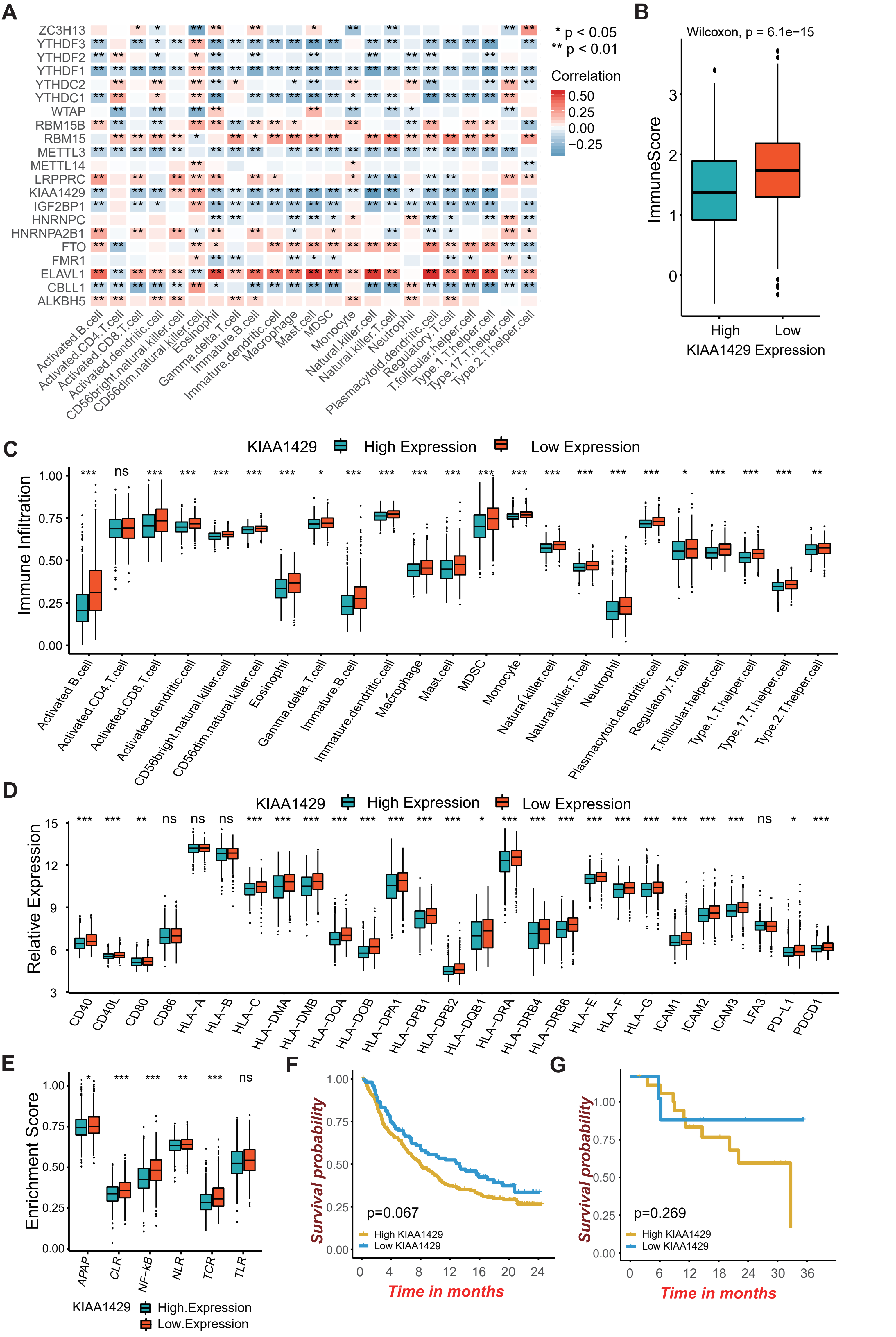

(A) The correlation between each TME infiltration cell type and each m⁶A regulator using spearman analyses. Negative correlation was marked with blue and positive correlation with red.(*P < 0.05; **P < 0.01) **(B)** Difference in immuneScore between KIAA1429 high expression and low expression. (p<0.0001, Wilcoxon test) **(C)** Difference in the abundance of each TME infiltrating cell between KIAA1429 high expression and low expression groups. **(D)** Difference in the expression of MHC molecules, costimulatory molecules, and adhesion molecule between KIAA 1429 high expression and low expression groups. The upper and lower ends of the boxes represented interquartile range of values. The lines in the boxes represented median value, and black dots showed outliers. The asterisks represented the statistical p value.(*P < 0.05; **P < 0.01; ***P < 0.001) **(E)** Differences in immune-activated pathways between KIAA 1429 high expression and low expression groups. APAR, antigen processing and presentation; CLR, C-type lectin receptor; NLR, NOD-like receptor; TCR, T cell receptor; TLR, Toll-like receptor.(*P < 0.05; **P < 0.01; ***P < 0.001) **(F,G)** Survival analyses for patients with low or high KIAA1429 expression in the anti-PD-L1 **(F)** and anti-PD-1 **(G)** immunotherapy cohorts using Kaplan-Meier curves.

Figure S4. Unsupervised clustering of 21 m^6A regulators in the ACRG gastric cancer cohort.

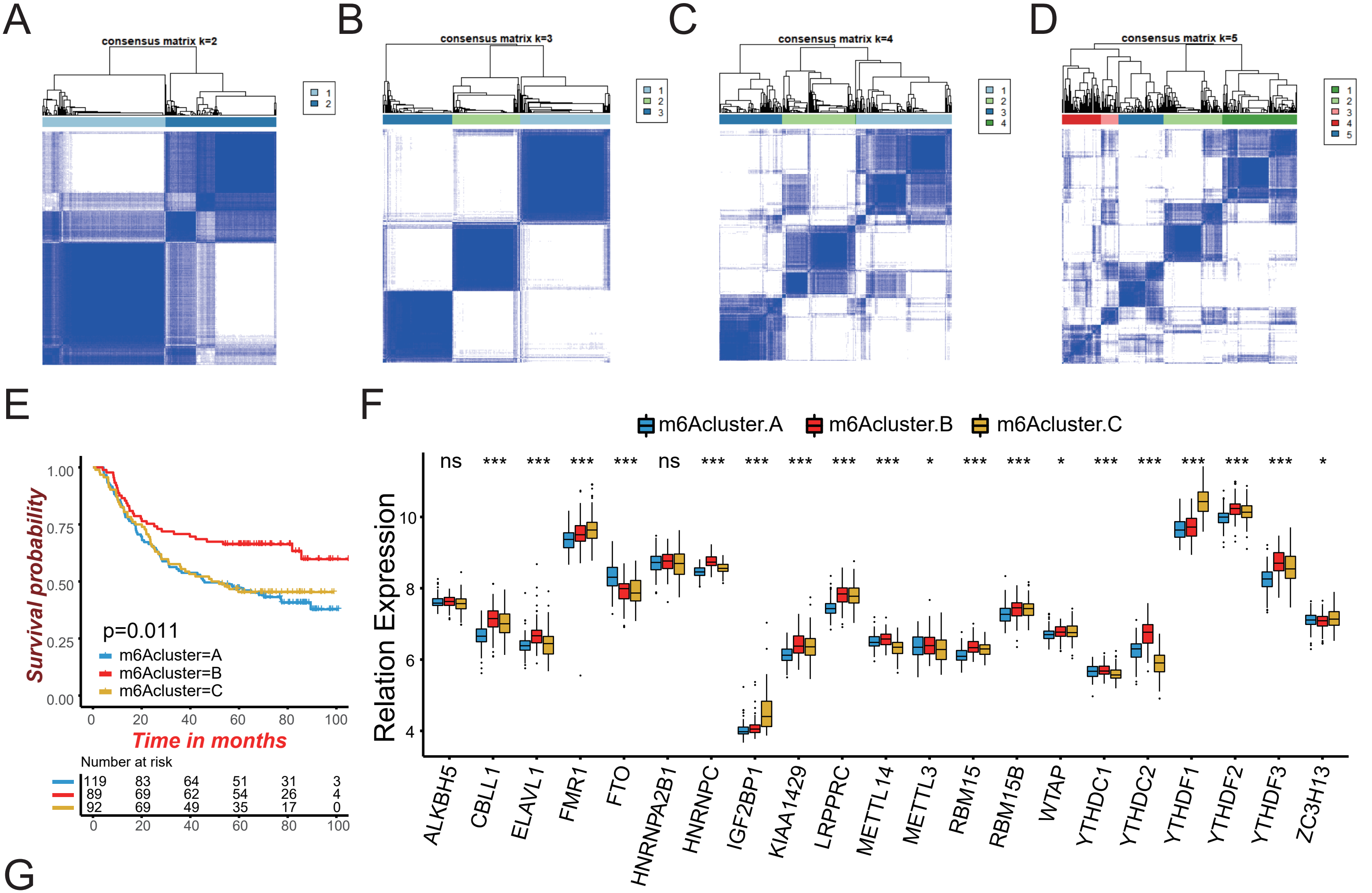

G

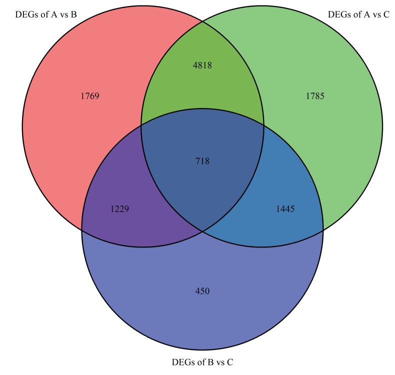

(A-D) Consensus matrices of the ACRG cohort for k = 2 - 5. **(E)** Survival analyses for the three m^6A modification patterns in ACRG cohort using Kaplan-Meier curves including 119 cases in m6Acluster-A, 89 cases in m6Acluster-B, and 92 cases in m6Acluster-C. The m6Acluster B showed significantly better overall survival than the other two m6Acluster.(p=0.011, Log-rank test) **(F)** The expression of 21 m^6A regulators in the three m6Aclusters. The upper and lower ends of the boxes represented interquartile range of values. The lines in the boxes represented median value, and black dots showed outliers. The asterisks represented the statistical p value.(*P < 0.05; **P < 0.01; ***P < 0.001) **(G)** 718 m^6A phenotype-related genes shown in venn diagram.

Figure S5. Characteristics of cytokine transcriptome, chemokine transcriptome and known signatures in distinct gene clusters

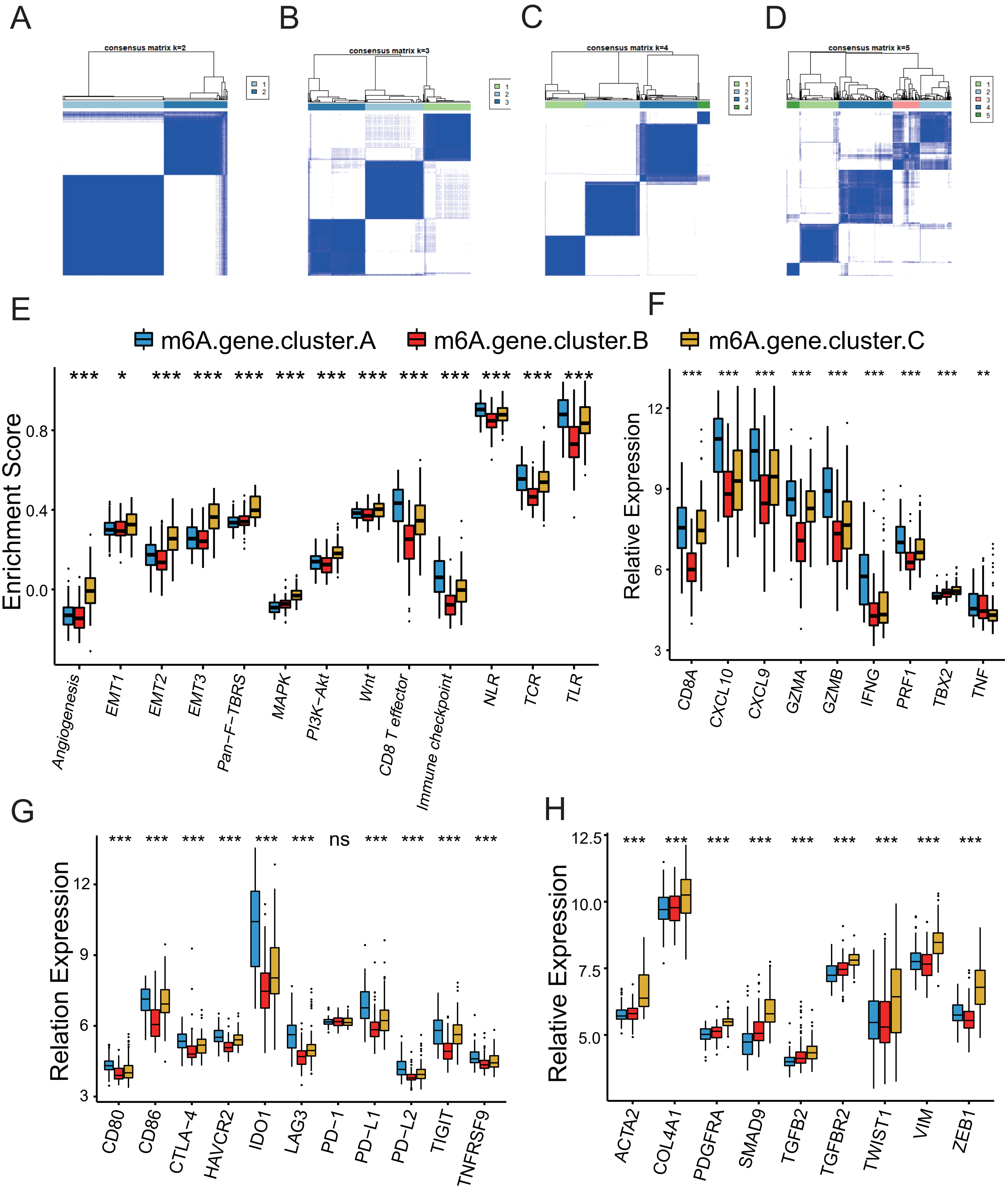

(A-D) Unsupervised clustering of 718 m⁶A phenotype-related genes in ACRG cohort and consensus matrices for k = 2 - 5. **(E)** Difference in the expression of known signatures including stromal-activation related signatures, tumor-promotion related signatures and immune-activation related signatures among three gene clusters. The expression of 21 m⁶A regulators in the three m6Aclusters. The upper and lower ends of the boxes represented interquartile range of values. The lines in the boxes represented median value, and black dots showed outliers. The asterisks represented the statistical p value.(*P < 0.05; **P < 0.01; ***P < 0.001) **(F)** Difference in the immune-activation related gene expression among three gene clusters. **(G)** Difference in the immune-checkpoint related gene expression among three gene clusters. **(H)** Difference in the TGFβ- EMT pathway-related gene expression among three gene clusters.

Figure S6. The prognostic value of m6Ascore and correlation between the clinicopathological features and m6Ascore.

A

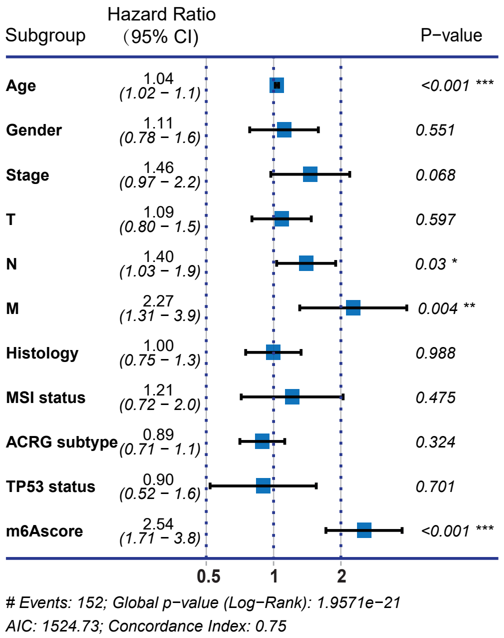

B

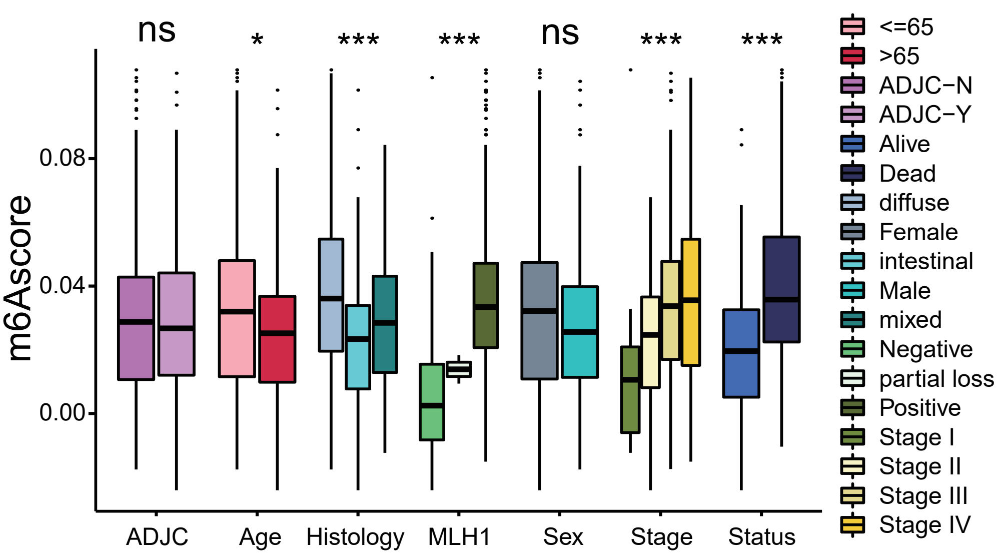

C

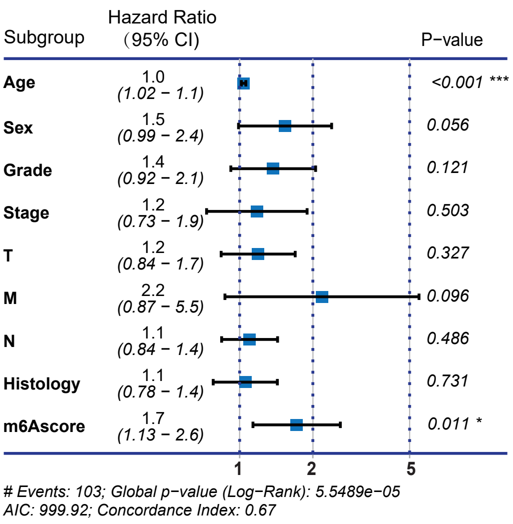

(A) Multivariate Cox regression analysis for m6Ascore in ACRG cohort shown by the forest plot. **(B)** Difference in m6Ascore among distinct clinical subgroups in ACRG cohort. ADJC, adjuvant chemotherapy. (*P < 0.05; **P < 0.01; ***P < 0.001) **(C)** Multivariate Cox regression analysis for m6Ascore in TCGA-STAD cohort shown by the forest plot.

Figure S7. The effect of microsatellite status and EB virus infection on the three m⁶A modification patterns and m⁶A regulators.

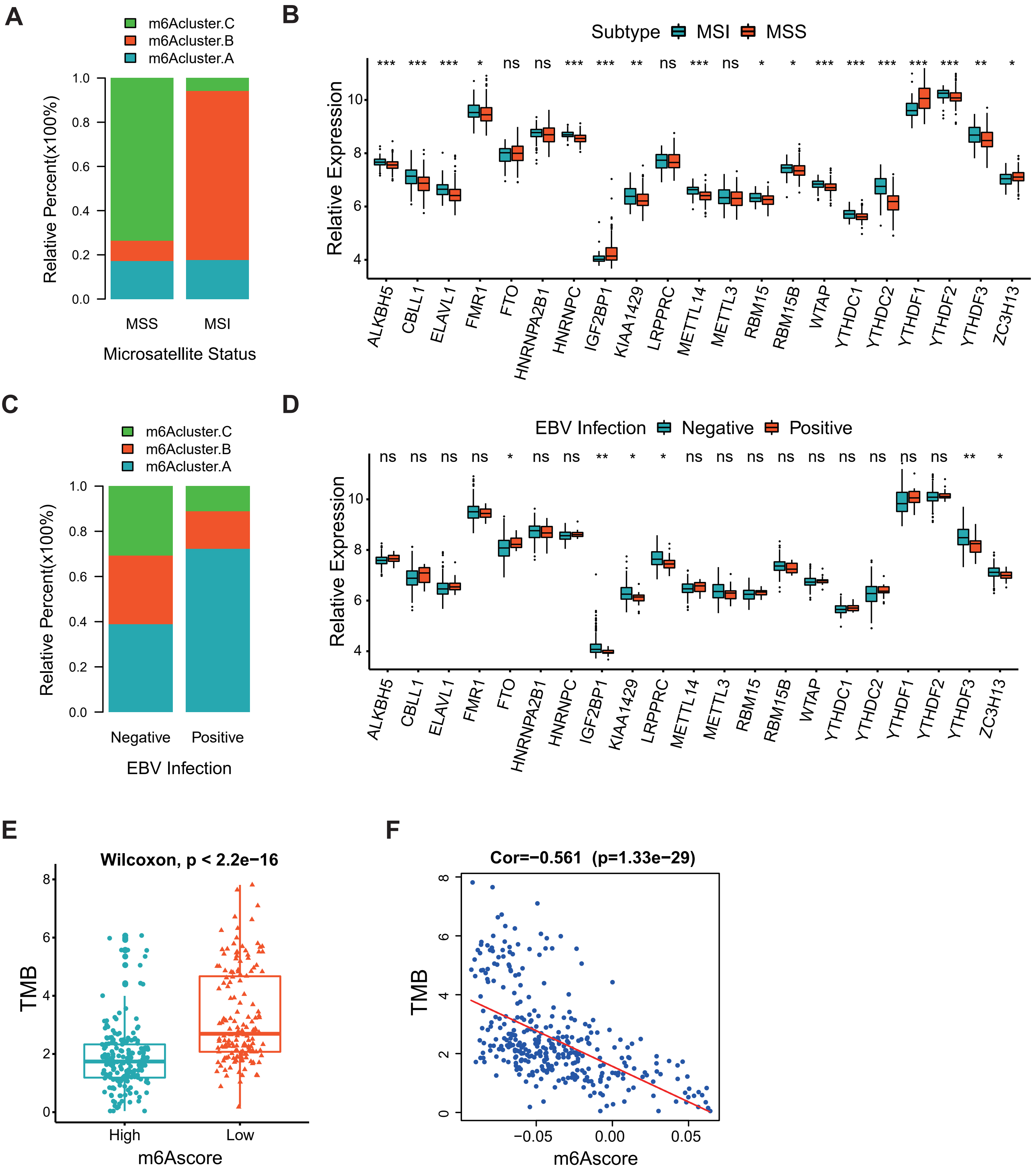

(A) The proportion of three m⁶A modification patterns in the MSI and MSS subtypes. MSI, microsatellite instability; MSS, microsatellite stable. **(B)** Difference in the expression of 21 m⁶A regulators between MSI and MSS subtypes. The upper and lower ends of the boxes represented interquartile range of values. The lines in the boxes represented median value, and black dots showed outliers. The asterisks represented the statistical p value.(*P < 0.05; **P < 0.01; ***P < 0.001) **(C)**The proportion of three m⁶A modification patterns in the EBV-positive and EBV-negative groups. **(D)** Difference in the expression of 21 m⁶A regulators between EBV-positive and EBV-negative groups. The asterisks represented the statistical p value.(*P < 0.05; **P < 0.01; ***P < 0.001)

Figure S8. Prognostic value of m6Ascore in gastric cancer cohorts and digestive cancer cohorts.

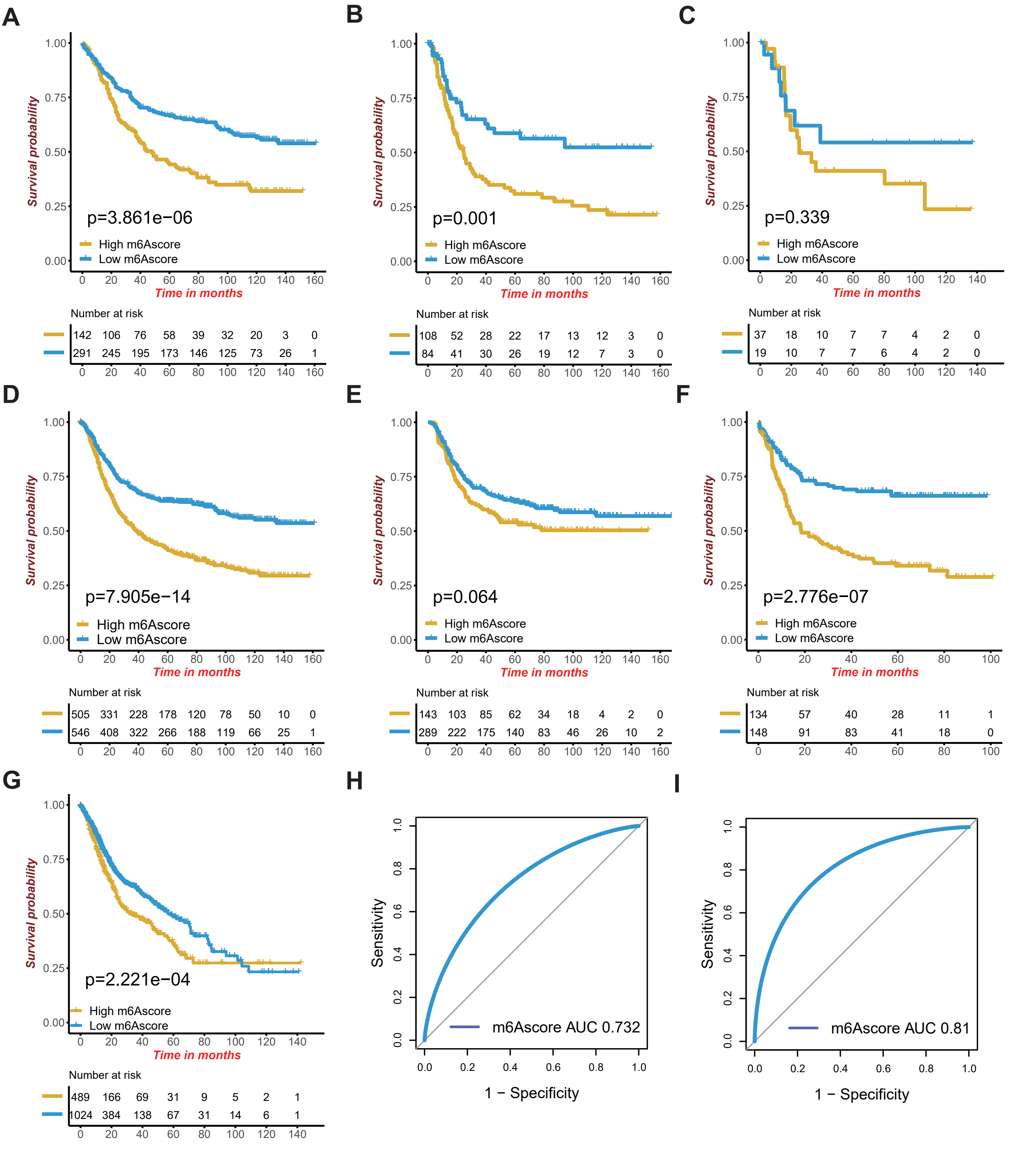

(A) GSE84437: HR,1.89; 95%CI 1.44 - 2.49. (P < 0.0001, Log-rank test)
(B) GSE15459: HR, 2.05; 95%CI 1.30 - 3.22. (P = 0.001, Log-rank test)
(C) GSE34942: HR, 1.52; 95%CI 0.64 - 3.63. (P = 0.339, Log-rank test)
(D) All GEO gastric cancer cohorts: HR, 1.94; 95%CI 1.62 - 2.31; (P < 0.0001, Log-rank test)
(E) Relapse-free survival analysis of m6Ascore in GSE26253 cohort. HR, 1.33; 95%CI 0.98 - 1.80. (P = 0.064, Log-rank test)
(F) Relapse-free survival analysis of m6Ascore in GSE62254 cohort. HR, 2.53; 95%CI 1.75 - 3.65. (P < 0.0001, Log-rank test)
(G) Survival analysis of m6Ascore in all digestive cancer cohorts from TCGA including cholangiocarcinoma, colon adenocarcinoma, pancreatic adenocarcinoma, esophageal carcinoma and liver hepatocellular carcinoma. HR, 1.4; 95%CI 1.17 - 1.68. (P < 0.001, Log-rank test)
(H) The predictive value of m6Ascore in gastric cancer cohorts. AUC, 0.732.
(I) The predictive value of m6Ascore in older patients with gastric cancer. AUC 0.81.
